# A systematic review of blood biomarkers with individual participant data meta-analysis of matrix metalloproteinase-7 in idiopathic pulmonary fibrosis

**DOI:** 10.1183/13993003.01612-2021

**Published:** 2022-04-28

**Authors:** Fasihul A. Khan, Iain Stewart, Gauri Saini, Karen A. Robinson, R. Gisli Jenkins

**Affiliations:** 1 Division of Respiratory Medicine, School of Medicine, University of Nottingham, Nottingham, UK; 2 Nottingham Biomedical Research Centre, National Institute for Health Research, Nottingham, UK; 3 Margaret Turner Warwick Centre for Fibrosing Lung Disease, National Health and Lung Institute, Imperial College London, London, UK; 4 Epidemiology and Health Policy and Management, Johns Hopkins University, Baltimore, MD, USA

## Abstract

**Background:**

Blood-derived biomarkers have been described extensively as potential prognostic markers in idiopathic pulmonary fibrosis (IPF), but studies have been limited by analyses using data-dependent thresholds, inconsistent adjustment for confounders and an array of end-points, thus often yielding ungeneralisable results. Meta-analysis of individual participant data (IPD) is a powerful tool to overcome these limitations. Through systematic review of blood-derived biomarkers, sufficient studies with measurements of matrix metalloproteinase (MMP)-7 were identified to facilitate standardised analyses of the prognostic potential of this biomarker in IPF.

**Methods:**

Electronic databases were searched on 12 November 2020 to identify prospective studies reporting outcomes in patients with untreated IPF, stratified according to at least one pre-specified biomarker, measured at either baseline, or change over 3 months. IPD were sought for studies investigating MMP-7 as a prognostic factor. The primary outcome was overall mortality according to standardised MMP-7 z-scores, with a secondary outcome of disease progression in 12 months, all adjusted for age, gender, smoking and baseline forced vital capacity.

**Results:**

IPD was available for nine studies out of 12 identified, reporting outcomes from 1664 participants. Baseline MMP-7 levels were associated with increased mortality risk (adjusted hazard ratio 1.23, 95% CI 1.03–1.48; I^2^=64.3%) and disease progression (adjusted OR 1.27, 95% CI 1.11–1.46; I^2^=5.9%). In limited studies, 3-month change in MMP-7 was not associated with outcomes.

**Conclusion:**

IPD meta-analysis demonstrated that greater baseline MMP-7 levels were independently associated with an increased risk of poor outcomes in patients with untreated IPF, while short-term changes did not reflect disease progression.

## Abstract 2

**Robust methodology using individual participant data meta-analysis demonstrates that baseline MMP-7 levels predict overall mortality and disease progression in patients with untreated IPF independent of age, gender, smoking status and lung function** https://bit.ly/2WlPudQ

## Introduction

Idiopathic pulmonary fibrosis (IPF) is a chronic progressive fibrotic lung disease of unknown aetiology that affects ∼3 million people worldwide, with a rising incidence and a median survival from diagnosis of ∼3 years [1–5]. Disease trajectory is variable, ranging from slow progression to rapid loss of lung function and death [6]. The most recognised biomarker of disease progression in IPF is the change in forced vital capacity (FVC) at 12 months [7, 8]. However, lung function measurements have limitations, including test variability related to patient effort and confounding effects of comorbidities such as emphysema [9].

Blood-derived biomarkers have been described extensively as potential prognostic markers that reflect disease severity, although none have been implemented into routine clinical practice. Studies of biomarkers have been limited by small sample sizes, inconsistent methodologies including inconsistent adjustment for confounding variables, a variety of end-points and analysis of outcomes using data-dependent biomarker thresholds, thus often yielding inconsistent and ungeneralisable results [10, 11].

Individual patient data (IPD) meta-analyses are considered the gold standard for collecting and synthesising evidence, offering a number of advantages over traditional aggregate methods, by enabling standardisation of analyses and outcomes, consistent adjustment for potential confounding factors and robust subgroup analyses according to patient characteristics [12, 13]. No published studies have utilised IPD to systematically synthesise the evidence for blood biomarkers in IPF. Through systematic review of blood-derived biomarkers, sufficient studies with measurements of matrix metalloproteinase (MMP)-7 were identified to facilitate standardised analyses of the prognostic potential of this biomarker in IPF. Thus, we explore the association between MMP-7 measured at baseline and change over 3 months, and clinical end-points including mortality and disease progression in adult patients with untreated IPF.

## Methods

The systematic review was conducted in accordance with a pre-specified protocol (PROSPERO registration number: CRD42019120402) and has been reported using PRISMA-IPD (Preferred Reporting Items for Systematic Reviews and Meta-Analyses of Individual Participant Data) guidelines [14].

### Search strategy and study selection

Electronic database searches were carried out in MEDLINE (1946 to latest), Embase (1974 to latest), Google Scholar, the Cochrane Central Register of Controlled Trials and ClinicalTrials.gov, with the last search carried out on 12 November 2020. Keywords and controlled vocabulary terms for “idiopathic pulmonary fibrosis” and “biomarkers”, alongside search filters for prognostic studies were applied (supplementary figure S1) [15]. Hand searches of reference lists in retrieved articles were conducted to identify further studies. Unpublished and ongoing studies were identified by searching preprint servers including medRxiv, bioRxiv and Wellcome Open Research. Following searches, two reviewers screened through titles and abstracts independently before full-text review. Disagreements were resolved by consensus with a third reviewer.

The review included all original prospective observational studies that reported outcomes in stable or exacerbating patients aged >18 years with antifibrotic naïve IPF, diagnosed according to contemporaneous consensus guidelines [16–18], stratified according to at least one pre-identified blood biomarker. Conference abstracts reporting sufficient detail were eligible for inclusion. Retrospective studies, case reports, animal studies and studies investigating non-IPF interstitial lung disease (ILD) were excluded. Language or year of publication restrictions were not applied. No minimal study sample size was specified for inclusion.

Studies reporting the following biomarkers measured at either baseline and/or trends over 3 months were eligible for review: biomarkers of epithelial dysfunction (MMP-7, Krebs von den Lungen-6, surfactant protein-A and -D, MMP-1, cancer antigen 125 (CA-125), carbohydrate antigen 19-9, vascular endothelial growth factor and insulin-like growth factor binding protein 2); biomarkers of extracellular matrix modelling (collagen synthesis peptides, neoepitopes, lysyl oxidase-like 2, periostin and osteopontin); and biomarkers of immune dysregulation (C-C motif chemokine ligand-18, chemokine ligand 13, interleukin-8, heat shock protein 70, chitinase-3-like protein 1 (YKL40) and intracellular adhesion molecule-1).

### Data extraction and risk-of-bias assessment

IPD were sought from corresponding authors of studies investigating MMP-7 as a prognostic factor, using secure and encrypted electronic mail communication. A minimum of three reminders were sent, each 4 weeks apart. Data from sponsored clinical studies were requested through various online portals (www.clinicalstudydatarequest.com, www.vivli.org, https://yoda.yale.edu). Requested data included participant demographics (age, gender, smoking status and baseline lung function), baseline and 3-month MMP-7 levels and outcomes including 12-month lung function and overall mortality (supplementary figure S2).

Where IPD were not made available, aggregate data were extracted from study publications using a proforma, and verified by a second reviewer. Data included study design, participant and biomarker characteristics and outcome data including sample sizes, mean values and standard deviations of biomarkers in individuals with and without the event. Time-to-event data were collected using adjusted hazard ratios (HR) where reported.

Risk-of-bias assessment was carried out independently by two reviewers using the Quality in Prognostic Studies (QUIPS) tool [19]. The QUIPS tool assesses the risk of bias across six domains: study participation, study attrition, prognostic factor measurement, outcome measurement, study confounding and statistical analysis and reporting. All studies were included in the review irrespective of their risk of bias rating. The GRADE (Grading of Recommendations, Assessment, Development and Evaluations) framework was applied to rate the overall quality of evidence for each outcome [20].

### Statistical analysis

All identified studies were included in the data synthesis, with summary tables for study characteristics. Multiple cohorts within the same study were treated as individual cohorts. The primary outcome was overall mortality. Secondary outcomes measures included change in percentage predicted FVC from baseline at 12 months and disease progression defined as 10% relative decline in FVC or death within 12 months of baseline. Hazard ratios for MMP-7 levels in predicting mortality, and odds ratios for predicting disease progression were estimated using a two-stage IPD meta-analysis with random effects and presented as forest plots. Estimates were adjusted for *a priori* confounders including age, sex, smoking history and baseline FVC. Unadjusted analyses are presented in the supplementary material (supplementary figure S10). Studies with a follow-up duration >3 years were censored for survival analyses. To standardise biomarker values across studies, z-scores specific to each study were calculated and analysed as exposure variables. The change in MMP-7 over 3 months was calculated where available using relative percentage change from baseline. Participants with missing data were excluded using listwise deletion. The I^2^ statistic was used to evaluate statistical heterogeneity between studies. Meta-regression was conducted where sufficient studies were included to explore variability in heterogeneity according to study design (cohort *versus* randomised trial), single-centre studies, non-peer-reviewed manuscripts, assay methods (ELISA *versus* non-ELISA) and the type of blood samples used (serum *versus* plasma). Publication bias was assessed using funnel plot analysis and Egger's test [21]. All statistical analyses were performed using Stata 16 (StataCorp, College Station, TX, USA). Due to methodological heterogeneity, marked difference in outcome measures and insufficient studies for IPD, biomarkers other than MMP-7 have been described narratively and in tables.

## Results

**FIGURE 1:**
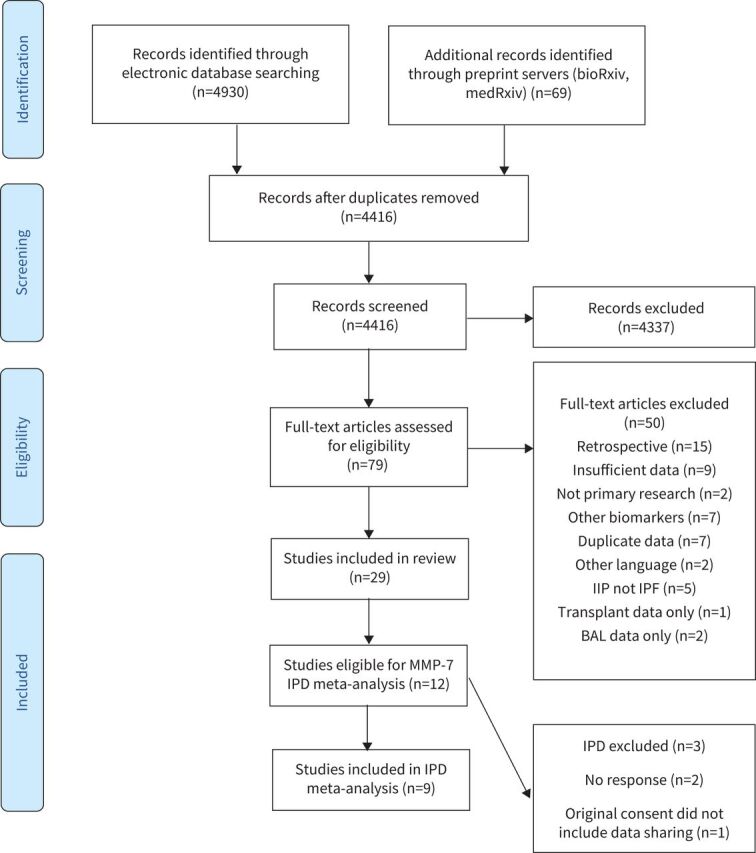
Flow diagram illustrating systematic search and screening strategy, including numbers of studies meeting eligibility criteria and numbers excluded. MMP: matrix metalloproteinase; IPD: individual participant data; IIP: idiopathic interstitial pneumonia; IPF: idiopathic pulmonary fibrosis; BAL: bronchoalveolar lavage.

Searches of the electronic databases on 12 November 2020 yielded 4930 articles, with a further 69 studies identified through preprint servers. Following the removal of duplicates, screening and full-text review, 29 studies published worldwide between 2007 and 2020 were included, reporting outcomes from 3950 IPF participants (figure 1). 12 studies reported outcomes in relation to MMP-7, of which IPD were available for nine (75%) studies, reporting data from 11 individual cohorts and 1664 participants (table 1). No issues with the integrity of IPD were identified. A further 15 blood biomarkers were evaluated across the included studies, with a number of studies evaluating combinations of biomarkers (supplementary table S1).

**TABLE 1:** Methodological characteristics of matrix metalloproteinase (MMP)-7 studies with baseline participant characteristics and outcome data

**First author, year [ref.]**	**Included in IPD meta-analysis**	**Country of study**	**IPF sample size**	**Study follow-up, months**	**Age, years**	**Male, %**	**Baseline FVC, % predicted**	**Baseline *D*_LCO_, % predicted**	**Relevant outcomes reported**
**Bauer, 2017 [30]**	No	Multinational	211 (BUILD-3 [42])	NR	63.1±8.9	64	75.7±10.7	47.7±10.7	Disease progression (FVC ≥10% decline, *D*_LCO_ ≥15%, acute exacerbation or death) up to end of study, change in FVC at 4 months
**Hamai, 2016 [35]**	Yes	Japan single centre	65	28 (16–45)	69.3±8.6	77	75.6±21.9	47.1±15.8	5-year mortality
**Maher, 2017 [27]**	Yes	UK multicentre	106 (discovery)	15 (15–15)	70.8±8.3	78	79±18.9	43.3±14.8	Overall mortality, disease progression at 12 months (all-cause mortality or FVC decline ≥10%)
	Yes		200 (validation)	15 (15–15)	72.5±7.7	76	81.4±19.2	49±16.9	
**Navaratnam, 2014 [36]/Clynick, 2020 [37] ^#^**	Yes	UK multicentre	205	42 (20–60)	73.2±8.7	74	84.7±18.7	43.7±15.8	Overall mortality, disease progression at 12 months (all-cause mortality or >10% FVC decline)
**Neighbors, 2018 [26]**	Yes	Multinational	221 (CAPACITY [43])	18 (17–21)	66.9±7.4	72	73.4±13.4	46.5±9.4	At 12 months: disease progression (FVC ≥10% absolute decline or death), change in FVC, death
	Yes		244 (ASCEND [44])	12 (11–12)	67.7±7.2	77	68.3±10.9	43.9±11.9	
**Oldham** **, 2019 [38]**	Yes	USA multicentre	199	19 (8–32)	71.5±8.9	74	68.5±19.1	48.5±20.4	24-month transplant-free survival, overall mortality
**Peljto, 2013 [23]**	No	Multinational	438 (INSPIRE [45])	19 (14–25)	66.6±7.5	74	72.2±12.4	47.3±8.9	Overall mortality
**Raghu, 2018 [39]**	Yes	Multinational	154	12 (12–12)	67.9±8.4	64	71.5±19.6	40.9±15.9	Disease progression at 52 weeks (FVC decrease ≥10% predicted or *D*_LCO_ decrease >15% or lung transplantation or death)
**Richards, 2012 [40]**	No	USA single centre	140 (derivation)	22±19	67.2±8.3	72	62±19.6	44.8±17.1	Overall mortality, disease progression (FVC relative decline ≥10% within any 1 year of follow-up)
	Yes		97 (validation)	42 (14–60)	68±8.7	66	60.8±17	45.4±19	
**Rosas, 2018 [25]**	Yes	USA multicentre	58	11 (11–12)	67.6±7.3	81	71.1±15.6	41.5±13.9	Change in FVC
**Sokai, 2015 [24]**	No	Japan single centre	57	15 (0.4–61)^¶^	69.4±8.5	90	84.2±21.3	43.7±14.2	Overall mortality, disease progression (death, FVC decline ≥10%, *D*_LCO_ ≥15% decline, admission due to respiratory failure) at 6 months
**Tzouvelekis, 2017 [41]**	Yes	USA single centre	97	17 (8–17)	70±8	79	70.2±16.5	47.2±16.9	Overall mortality, disease progression (FVC decline >10% predicted over study period)

Data are presented as mean±sd or median (interquartile range), unless otherwise stated. IPD: individual participant data; IPF: idiopathic pulmonary fibrosis; FVC: forced vital capacity; *D*_LCO_: diffusing capacity of the lung for carbon monoxide; NR: not reported. ^#^: *post hoc* analysis (Clynick *et al.* [37]) of Navaratnam *et al.* [36]. Original study did not report biomarker data. ^¶^: median (range).

**FIGURE 2:**
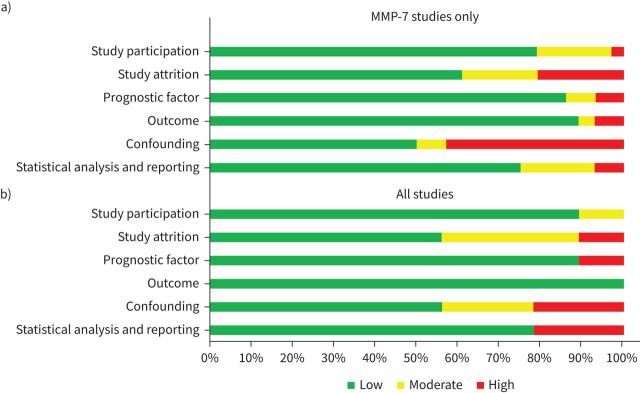
Risk of bias assessment for a) matrix metalloproteinase (MMP)-7 studies only; b) all included studies. The risk of bias across studies was rated as low, moderate or high in six categories using the Quality in Prognostic Studies tool.

Risk-of-bias assessment of the retrieved studies identified limitations and a number of possible biases (figure 2, supplementary table S2). For studies included in the MMP-7 meta-analysis, publication bias was not detected statistically, but visual inspection of funnel plots suggested that publication bias was present for some of the outcomes assessed (supplementary figures S3 and S4). Most MMP-7 studies defined the study population specifically with clear inclusion/exclusion criteria. Biomarkers were measured consistently using the same sample matrices (plasma or serum) across included participants in each study, although details of assay platforms used to measure the analytes were frequently unreported. Outcome data were measured objectively and applied consistently to all study participants. Studies evaluating biomarkers other than MMP-7 had similar limitations and risks of bias. Blood biomarkers are known to be influenced by age and sex, as well as possible lifestyle factors such as smoking, which along with baseline lung function are all confounders upon disease outcome [22]. In approximately half of all included studies, possible confounders were not measured, and there was inconsistent adjustment in estimations where accepted confounders were measured. Moreover, in a number of studies, analyses were performed using data-dependent biomarker thresholds that were inconsistent across studies.

### Association between blood biomarkers and clinical outcomes

#### Baseline blood biomarkers that predict mortality

**FIGURE 3:**
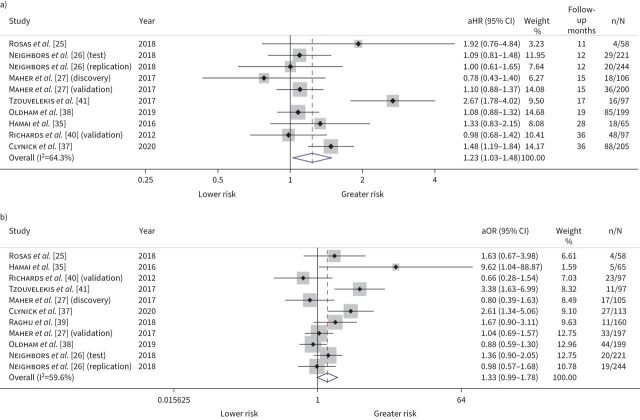
Mortality forest plot. a) Overall mortality; b) mortality at 12 months. Adjusted effect sizes with 95% confidence intervals per standard deviation increase in baseline matrix metalloproteinase-7. aHR: adjusted hazard ratio; aOR: adjusted odds ratio. All estimates were adjusted for age, sex, smoking status and baseline forced vital capacity. Weights are from random-effects model.

10 studies evaluated the relationship between mortality and MMP-7, with IPD available for eight studies totalling 1492 participants. Meta-analysis demonstrated that greater baseline MMP-7 values were associated with a 23% increased risk of overall mortality (adjusted (a)HR 1.23 per standard deviation increase, 95% CI 1.03–1.48; I^2^=64.3%) (figure 3a), although there was substantial statistical heterogeneity which could not be explained by variability in the factors assessed (supplementary table S3). When mortality at 12 months was examined specifically, baseline MMP-7 levels were inconclusively associated with death (aHR 1.33 per standard deviation increase, 95% CI 0.99–1.78; I^2^=59.6%) (figure 3b). Applying the GRADE framework, we rate the confidence in mortality estimates with moderate certainty (supplementary table S4). Where IPD were unavailable, MMP-7 values >5.7 ng·mL^−1^ were associated with increased mortality (aHR 2.18, 95% CI 1.1–4.32) over a median follow-up of 19 months in a study of 438 participants [23]. A further study of 57 participants found that MMP-7 levels did not predict death [24] (supplementary table S5).

**FIGURE 4:**
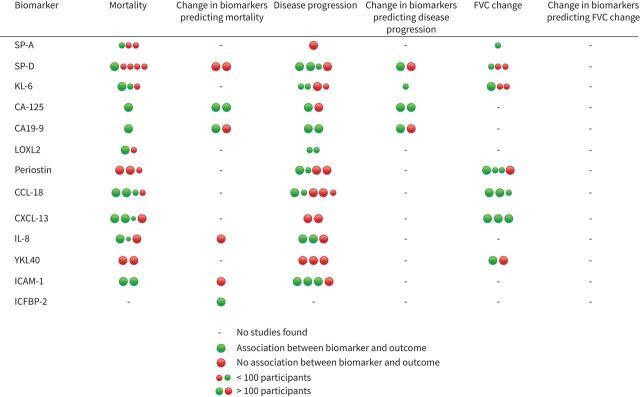
Summary of study results. Each dot represents a study (or individual cohort in studies with more than one cohort). FVC: forced vital capacity; SP: surfactant protein; KL: Krebs von den Lungen; CA-125: cancer antigen 125; CA19-9: carbohydrate antigen 19-9; LOXL: lysyl oxidase-like; CCL: C-C motif chemokine ligand; CXCL: chemokine ligand; IL: interleukin; YKL40: chitinase-3-like protein 1; ICAM: intracellular adhesion molecule; IGFBP: insulin-like growth factor binding protein.

The primary outcome of mortality was evaluated for a further 14 biomarkers in a total of 17 studies not assessed in IPD meta-analysis, with inconsistent and inconclusive findings (figure 4 and supplementary table S5). Study follow-up times were inconsistent, effect sizes varied with wide confidence intervals, and estimates were often unadjusted for important covariates.

#### Change in biomarkers predicting mortality

Three studies totalling 498 participants explored the association between MMP-7 change over 3 months and mortality [25, 26]. IPD meta-analysis showed no association with mortality (aHR 1.00, 95% CI 0.99–1.02; I^2^=53.3%), nor when mortality was censored at 12 months (aOR 1.00, 95% CI 0.99–1.01; I^2^=37.4%) (supplementary figures S5 and S6).

Three publications from the same cohort evaluated the relationship between longitudinal biomarker measurement and mortality [27–29]. In both discovery and validation cohorts, a rise in CA-125 over 3 months doubled the risk of death, but the remaining biomarkers were not predictive of mortality (figure 4 and supplementary table S6). A validation cohort of 145 participants demonstrated replication of rising neoepitopes degraded by MMPs (C1M, C3M, C6M and CRPM), but the rate of change of collagen synthesis peptides was not associated with mortality [29].

#### Baseline biomarkers that predict disease progression and change in FVC

**FIGURE 5:**
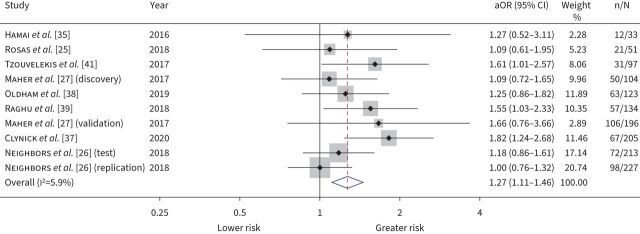
Disease progression forest plot. Pooled adjusted odds ratios (aOR) with 95% confidence intervals for risk of disease progression, per standard deviation increase in baseline matrix metalloproteinase-7. All estimates were adjusted for age, sex, smoking status and baseline forced vital capacity. Weights are from random-effects model.

**FIGURE 6:**
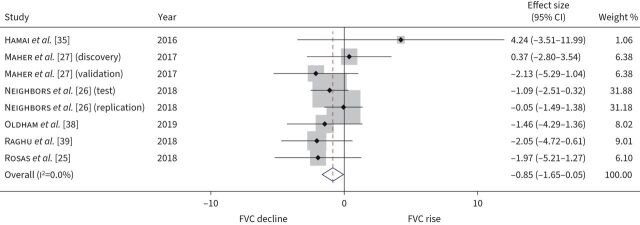
Relative change in percentage predicted forced vital capacity (FVC) forest plot. Pooled effect size with 95% confidence intervals for FVC % pred relative change at 12 months, per standard deviation increase in baseline matrix metalloproteinase-7. All estimates were adjusted for age, sex, smoking status and baseline FVC. Weights are from random-effects model.

10 studies measured MMP-7 levels as markers of disease progression, with eight studies totalling 1383 participants included in the IPD meta-analysis. Meta-analysis demonstrated that baseline MMP-7 was associated with disease progression (aOR 1.27 per standard deviation increase, 95% CI 1.11–1.46; I^2^=5.9%) (figure 5). While heterogeneity was low, meta-regression identified sample assay techniques (ELISA *versus* other) to be a source of heterogeneity. In subgroup analysis according to assay, the odds ratio for disease progression was estimated at 1.56 per standard deviation increase (95% CI 1.26–1.82; I^2^=0%) when restricted to studies using ELISA (supplementary figure S7). When the relationship between baseline MMP-7 and relative change in FVC at 12 months was examined specifically in six studies of 891 participants, meta-analysis indicated that a 1 sd greater baseline MMP-7 was associated with a −0.85% relative change in 12-month percentage predicted FVC (95% CI −1.65–−0.05%; I^2^=0%) (figure 6). We assess findings for disease progression and change in FVC outcomes with high certainty (supplementary table S4). For studies not included in IPD meta-analysis, baseline MMP-7 values >3.8 ng·mL^−1^ doubled the risk of disease progression (aHR 2.2, 95% CI 1.4–3.7) over a median follow-up of 19 months in 211 participants [30]. In a further study of 57 participants, MMP-7 did not predict disease progression (supplementary table S7).

Disease progression was evaluated for a number of other biomarkers in 19 studies that were not included in IPD meta-analysis. None were consistently predictive of disease progression, although there was significant heterogeneity in adopted definitions of disease progression, with lung function indices, mortality, transplant and acute exacerbations included in various combinations at nonunified time points (figure 4 and supplementary tables S7 and S8).

#### Change in biomarkers predicting disease progression

Three studies totalling 481 participants investigating the association between MMP-7 change over 3 months and disease progression were included in IPD meta-analysis. Change in MMP-7 over 3 months was not associated with disease progression (aOR 1.00 per percentage increase, 95% CI 0.99–1.01; I^2^=22.5%) (supplementary figure S8), nor with change in FVC over 12 months (effect size 0.01% increase per percentage MMP-7 increase, 95% CI −0.07–0.08; I^2^=60.8%) (supplementary figure S9). In a study of 211 participants not included in IPD meta-analysis, a two-fold change in MMP-7 over 4 months was associated with doubling the risk of disease progression [30].

In one study, participants with progressive disease had rising concentrations of CA-125 over 3 months compared to those with stable disease, but no relationship was replicated for other biomarkers [27] (figure 4, supplementary table S9).

## Discussion

This systematic review of prospective studies in patients with untreated IPF identified 16 blood-derived biomarkers and assessed six outcome variables, but there were only sufficient studies to undertake an IPD meta-analysis for MMP-7. IPD meta-analysis demonstrated that baseline MMP-7 levels predicted all-cause mortality and disease progression and correlated with percentage predicted FVC change over 12 months. There was a 23% greater risk of overall mortality and 27% greater risk of disease progression per standard deviation increase in baseline MMP-7 values. An inconclusive association was observed for risk of 12-month mortality. Notably, MMP-7 levels did not seem to change longitudinally over 3 months, with no association observed with any of the measured outcomes. However, a study not included in quantitative synthesis suggested that in those individuals where MMP-7 does rise, there may be an associated risk in progression [30]. Mortality outcomes were rated with moderate certainty and disease progression and change in FVC outcomes with high certainty (supplementary table S4).

Our IPD meta-analysis represents the first time it has been possible to synthesise blood biomarker findings in IPF. The meta-analysis was focused on MMP-7 as there were sufficient studies available; however, individually these had yielded inconsistent results, reported data-dependent thresholds and often had not adjusted for confounding factors. IPD enabled analysis of MMP-7 levels as continuous variables transformed to z-scores to overcome assay variability and supported standardised definition of outcomes and consistent adjustment for important covariates, which enabled robust and reliable conclusions. We performed two-stage IPD meta-analysis, which does not assess study estimate and effects simultaneously, although it is considered to produce unbiased estimates [31], and enabled modelling IPD from 1492 participants across separate secure servers and portals. Analysis of heterogeneity in IPD meta-analysis indicated that assay type was a significant contributor to heterogeneity, particularly in estimates of disease progression.

There are limitations to this review. While language restrictions were not applied, two articles in Japanese were excluded as they could not be translated to English to assess inclusion criteria. We included only those studies where participants were diagnosed according to international consensus guidelines, supporting the robustness and generalisability of our findings. We excluded studies in idiopathic interstitial pneumonias not specific to IPF, which limits interpretation in non-IPF ILDs, although ongoing studies exploring shared mechanistic pathways will provide further insight [32]. Furthermore, by focusing on untreated IPF patients, our results do not address the theranostic value of MMP-7 in relation to antifibrotic therapy. There was significant statistical heterogeneity in some of the outcomes, and therefore these should be interpreted with caution. We were unable to explain all the residual heterogeneity using the factors we assessed. IPD was not obtained from a limited number of suitable studies, and therefore we had to report these findings narratively.

Biomarkers of disease activity have the potential to facilitate clinical management and transform early-phase clinical trials by acting as surrogate end-points. Dysfunctional epithelial cells contribute to fibrogenesis by secreting profibrotic mediators including MMPs [33], responsible for degrading multiple components of extracellular matrix, activating biological mediators and facilitating epithelial–mesenchymal transition [34]. Further research could elucidate the relationship between IPF pharmacotherapy and MMP-7, particularly to identify whether changes in MMP-7 levels may represent a biomarker of therapeutic response. From a clinical perspective, MMP-7 should be considered for implementation as a prognostic tool at the point of diagnosis, especially where lung function testing is cumbersome or unavailable.

Due to heterogeneity in study designs and reported outcomes, there were insufficient data for quantitative analysis in non-MMP-7 studies. While many biomarkers showed an association with mortality in single studies, replication of effects across studies was weak. We highlight sources of considerable bias and variability. Studies were typically observational, of relatively modest size with a lack of pre-specified power calculations. A number of different laboratory techniques were applied to measure biomarker levels across studies, with very few studies reporting detailed assay information, particularly with regards to measures of precision, and there was inconsistency in thresholds defining positive and negative biomarker result. Short-term changes in biomarker concentrations over 3 months were often not associated with specified clinical outcomes, suggesting that further studies are needed before such biomarkers can be adopted clinically. Further biomarker research should focus on rigorously designed longitudinal studies with discovery and validation cohorts, using validated biomarker assays and standardised end-points. Furthermore, it is possible that combinations of biomarkers will add granularity to our understanding of pathogenesis and prognosis of IPF and further studies evaluating their utility are needed. As further studies are published, IPD meta-analysis should be considered to produce more reliable results and support generalisability.

In summary, while a number of other blood biomarkers have been studied for predicting prognosis, there is currently insufficient replication to enable adoption into clinical testing, with the possible exception of MMP-7. We apply robust methodology and IPD meta-analysis to demonstrate baseline MMP-7 levels predict overall mortality and disease progression in patients with untreated IPF independent of age, gender, smoking status and lung physiology. However, short-term changes in MMP-7 over 3 months offered limited prognostic value in the absence of an empirical threshold.

## Supplementary material

10.1183/13993003.01612-2021.Supp1 **Please note:** supplementary material is not edited by the Editorial Office, and is uploaded as it has been supplied by the author.
Supplementary material erj-01612-2021.supplement

## Shareable PDF

10.1183/13993003.01612-2021.Shareable1 This one-page PDF can be shared freely online.
Shareable PDF ERJ-01612-2021.Shareable
