# Supplementary material for: A systematic review of blood biomarkers with individual participant data meta-analysis of matrix metalloproteinase-7 in idiopathic pulmonary fibrosis
Source: Eur Respir J. 2022 Apr 28;59(4):2101612. doi: 10.1183/13993003.01612-2021 (PMC9202487; doi:10.1183/13993003.01612-2021)
Supplement: Supplementary file 1 [file erj-01612-2021.supplement.pdf]

Supplementary Material

Figure S1 - MEDLINE search strategy

Figure S2 – Letter to authors for individual participant data

Figure S3 – Funnel plots for baseline MMP-7

Figure S4 – Funnel plots for change in MMP-7 over 3 months

Figure S5 – Forest plot for change in MMP-7 over 3 months and overall mortality

Figure S6 – Forest plot for change in MMP-7 over 3 months and 12 months mortality

Figure S7 – Forest plot for baseline MMP-7 and disease progression separated by ELISA and non-ELISA

Figure S8 – Forest plot for change in MMP-7 over 3 months and disease progression

Figure S9 – Forest plot for change in MMP-7 over 3 months and relative change in FVC at 12 months

Figure S10 – Forest plot for baseline MMP-7 and mortality and disease progression in unadjusted analyses

Table S1 – Methodological characteristics of non-MMP7 studies

Table S2 – Risk of bias for included studies per individual study

Table S3 – Meta-regression for variables assessed

Table S4 – GRADE rating

Table S5 – Table of mortality outcomes for baseline biomarkers

Table S6 – Table of mortality outcomes for short term change in biomarkers

Table S7 – Table of disease progression definition and outcomes for baseline biomarkers

Table S8 – Table of FVC change definitions and outcomes for baseline biomarkers

Table S9 – Table of disease progression definitions and outcomes for short term change in biomarkers

Participants	Intervention	Intervention	Outcomes
1. idiopathic pulmonary fibros*.mp.	12. Mucin-1/	45. Chitinase-3-Like Protein 1/ or Chitinase-3-like protein 1.mp.	78. prognosis.sh.
2. pulmonary fibros*.mp.	13. KL-6.mp.	46. IGFBP-2.mp. or Insulin-Like Growth Factor Binding Protein 2/	79. diagnosed.tw.
3. Pulmonary Fibrosis/ or Idiopathic Pulmonary Fibrosis/	14. krebs von den lungen-6.mp.	47. Insulin like growth factor binding protein 2.mp.	80. cohort:.mp.
4. cryptogenic fibrosing alveolitis.mp.	15. SP-A.mp.	48. ICAM-1.mp. or Intercellular Adhesion Molecule-1/	81. predictor:.tw.
5. usual interstitial pneumonia*.mp.	16. Pulmonary Surfactant-Associated Protein A/	49. VEGF.mp. or Vascular Endothelial Growth Factor A/	82. death.tw.
6. Fibrosing alveolitis.mp.	17. Pulmonary Surfactant-Associated Protein D/	50. HSP70 HEAT-SHOCK PROTEINS/ or HSP70.mp.	83. exp models, statistical/
7. Idiopathic Interstitial Pneumonia*.mp.	18. Pulmonary Surfactants/	51. LEPTIN/ or Leptin.mp.	84. disease progression.sh.
8. Interstitial pneumonia*.mp.	19. SP-D.mp.	52. CXCL13.mp. [mp=title, abstract, original title, name of substance	85. disease progression.mp.
9. Idiopathic interstitial lung disease.mp.	20. surfactant protein*.mp.	53. Chemokine CXCL13/ or C-X-C motif chemokine 13.mp.	
10. Chronic interstitial pneumonia*.mp.	21. CA-125 Antigen/ or CA125.mp.	54. Forced Vital Capacity.mp. or Vital Capacity/	
	22. cancer antigen 125.mp.	55. FVC.mp.	
	23. mucin 16.mp.	56. Forced Expiratory Volume/ or FEV1.mp.	
	24. CA-19-9 Antigen/ or CA19-9.mp.	57. forced expiratory volume.mp.	
	25. cancer antigen 19-9.mp.	58. 6-minute walk.mp.	
	26. carbohydrate antigen 19-9.mp.	59. Six-minute walk.mp.	
	27. Matrix Metalloproteinase 1/ or MMP-1.mp.	60. Walk Test/	
	28. Matrix Metalloproteinase 7/ or MMP-7.mp.	61. walk test.mp.	
	29. matrix metalloproteinase.mp. or Matrix Metalloproteinases/	62. 6MWT.mp.	
	30. LOXL2.mp.	63. 6MWD.mp.	
	31. Protein-Lysine 6-Oxidase/	64. Pulmonary diffusing capacity.mp. or Pulmonary Diffusing Capacity/	
	32. protein-lysine 6-oxidase.mp.	65. Diffusion capacity for carbon monoxide.mp.	
	33. periostin.mp.	66. DLCO.mp.	
	34. Osteoblast-specific factor 2.mp.	67. Transfer factor.mp. or Transfer Factor/	
	35. Epitopes/ or Neoepitope*.mp.	68. Gas transfer.mp.	
	36. Chemokines, CC/ or CCL18.mp.	69. TLCO.mp.	
	37. Chemokine CCL18.mp.	70. KCO.mp.	
	38. Chemokines, CC/ or CC-chemokine ligand 18.mp.	71. PHYSIOLOGY/	
	39. IL-8.mp. or Interleukin-8/	72. Physiolog*.mp.	
	40. Interleukin-8.mp.	73. SPIROMETRY/	
	41. CXCL8.mp.	74. spiromet*.mp.	
	42. Chemokine ligand 8.mp.	75. biomarkers.mp. or BIOMARKERS/	
	43. Chitinase-3-Like Protein 1/ or YKL-40.mp.	76. ((Serum or clinical or immun* or lab or laboratory or biochemical or biological) and marker*).mp.	
	44. CHI3L1.mp.		

Supplementary Figure S1 – MEDLINE search strategy (last search carried out on 12th November 2020). “OR” was used to combine search terms within each PICOS category, with “AND” used to combine search terms across PICOS categories.

Copy of email sent to authors

We would be very grateful for your assistance in undertaking a robust meta-analysis. The team at University of Nottingham (UK), led by Prof Gisli Jenkins, are conducting a systematic review and meta-analysis of blood biomarkers in IPF. The protocol for the study can be found on PROSPERO: https://www.crd.york.ac.uk/prospero/display_record.php?RecordID=120402

As part of the review, we will conduct a meta-analysis of the association of MMP-7 levels with mortality in IPF. We have chosen this biomarker because there is sufficient published data to make it feasible and useful.

To assist with this, we would be extremely grateful if you could kindly provide us with individual patient data from your highly relevant study entitled "…" published in …

We also note significant heterogeneity in disease progression definitions across individual studies, and therefore hope to meta-analyse MMP-7 level associations with a shared definition based on FVC and mortality and would also appreciate data to assist with this. We appreciate the inconvenience such requests entail, and we would like to make the process as smooth as possible, we will of course acknowledge all support.

The attached excel spreadsheet highlights the anonymised data we are seeking for each patient, where available:

- MMP-7 level (baseline and 3-months)
- Assay method (type of assay used)
- Sample type (serum or plasma)
- Age
- Gender (M or F)
- Follow up time (days)
- Dead or alive at end
- Time to death (days)
- Baseline FVC (% predicted)
- 3-month FVC (% predicted)
- 12-month FVC (% predicted)
- Smoking (ever or never)

Thank you for your help, we look forward to communicating with you further.

Supplementary Figure S2 – copy of message sent to authors for individual participant data. A minimum of three reminders, 4 weeks apart were sent.

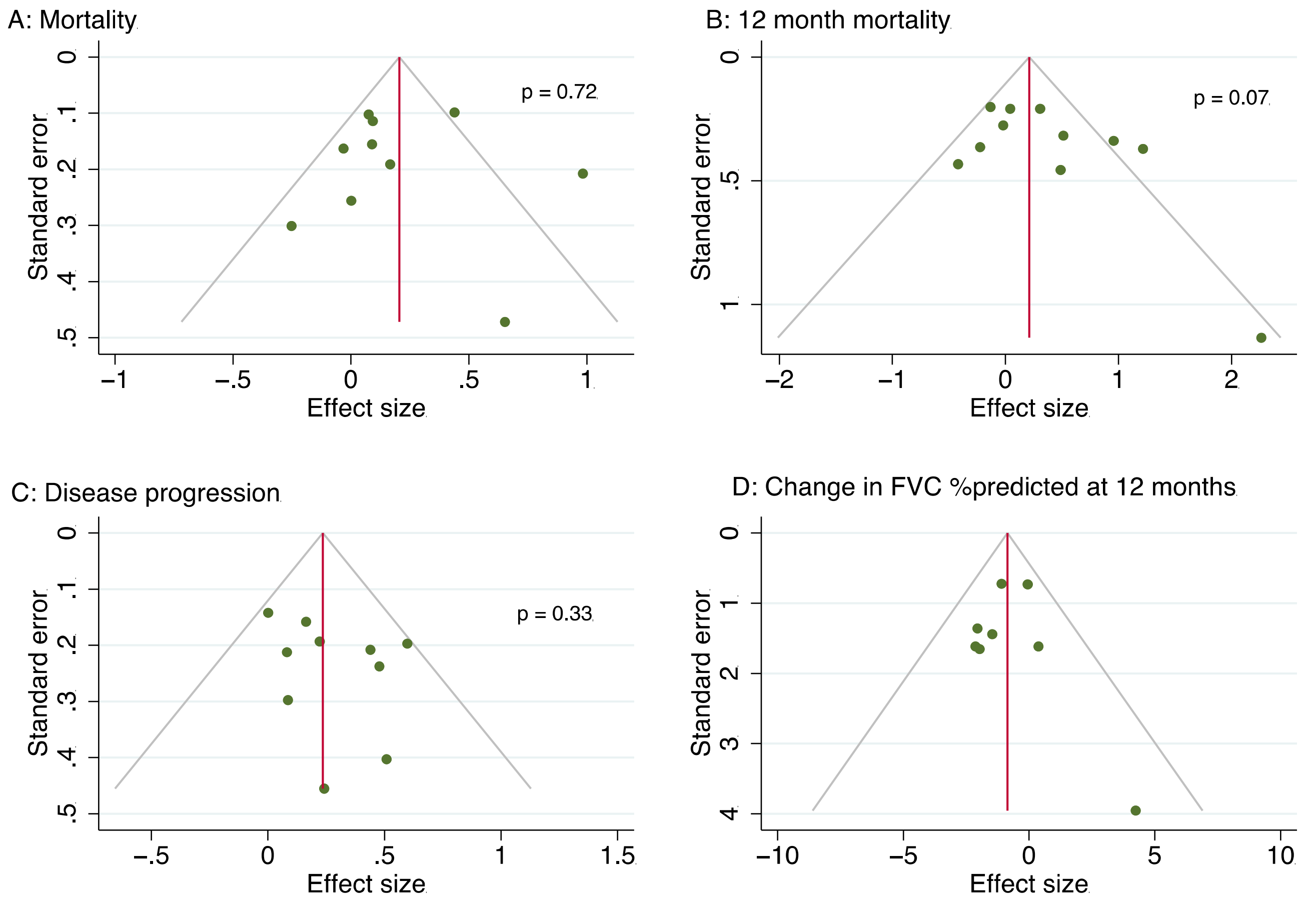

Supplementary Figure S3 – Funnel plots for outcomes evaluated in baseline MMP-7 IPD meta-analysis. A: overall mortality, B: 12-month mortality, C: Disease progression, D: Change in percent predicted FVC at 12 months. Publication bias assessed using Egger's test for outcomes with at least ten studies, and p values presented next to funnel plot.

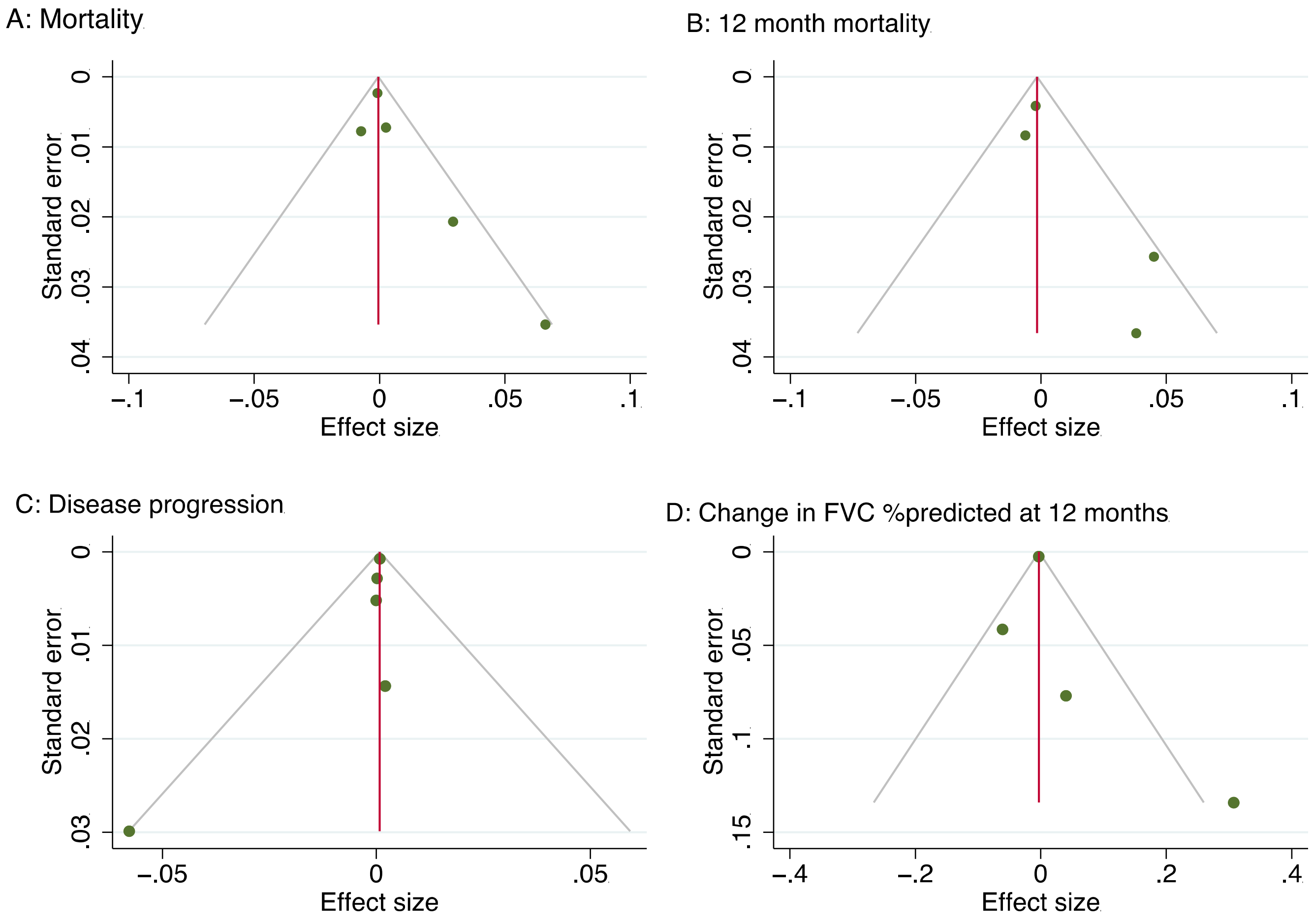

Supplementary Figure S4 – Funnel plots for outcomes evaluated for three-month change in MMP-7 IPD meta-analysis. A: overall mortality, B: 12-month mortality, C: Disease progression, D: Change in percent predicted FVC at 12 months.

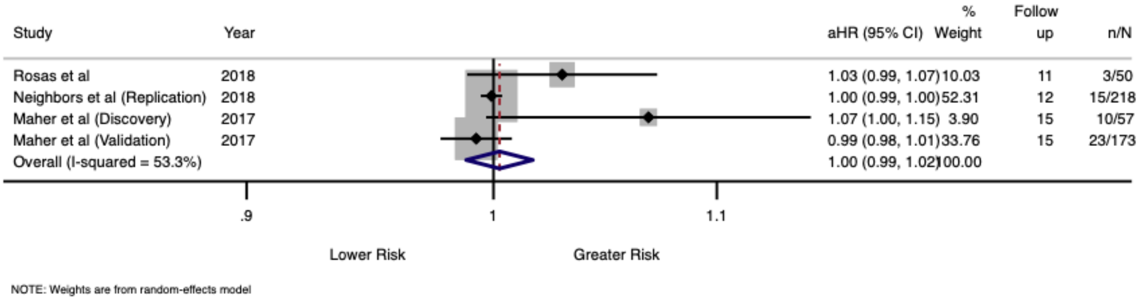

Supplementary Figure S5 - Pooled hazard ratios with 95% confidence intervals for risk of overall mortality, per percent relative increase in MMP-7 from baseline to three months. Study follow up time shown in months. n denotes the number of deaths, and N represents the total number of participants included per study.

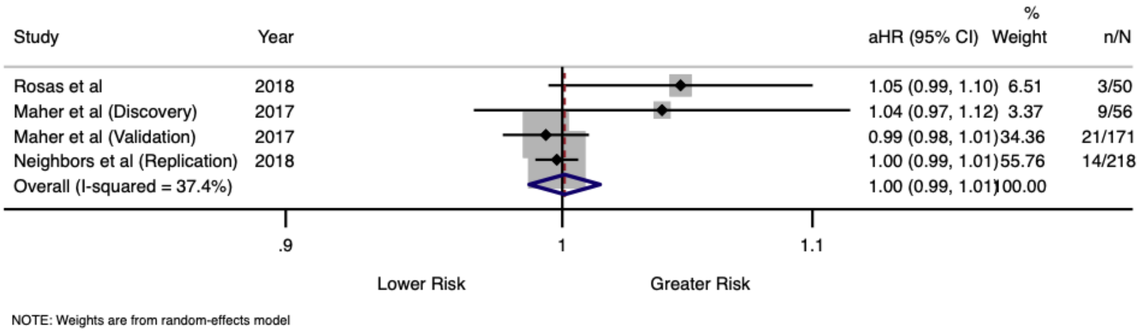

Supplementary Figure S6 - Pooled hazard ratios with 95% confidence intervals for risk of mortality at 12 months, per percent relative increase in MMP-7 from baseline to three months. n denotes the number of deaths, and N represents the total number of participants included per study.

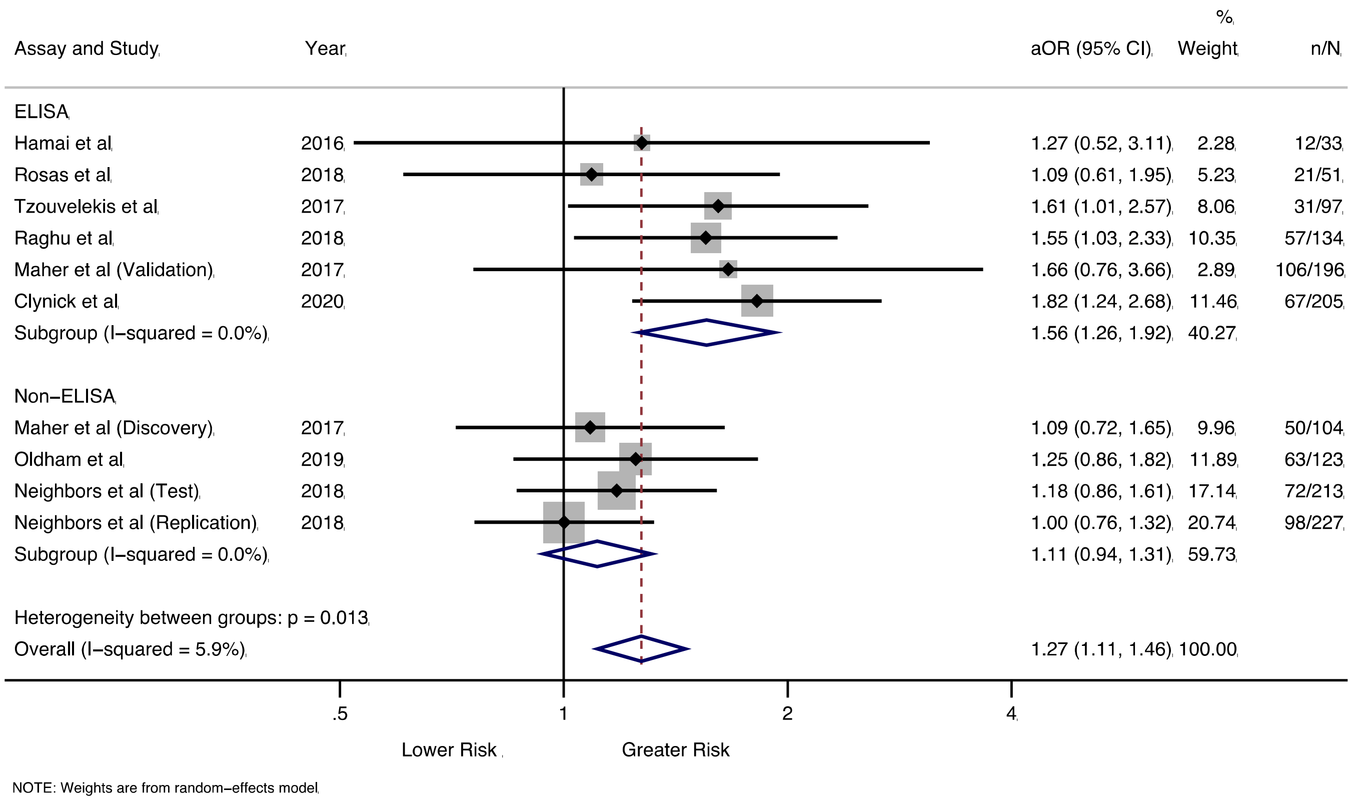

Supplementary Figure S7 – Pooled odds ratios with 95% confidence intervals for risk of disease progression, per standard deviation increase in baseline MMP-7. Separated by ELISA and non-ELISA measurements. n denotes the number of progressors, and N represents the total number of participants included in the analysis per study.

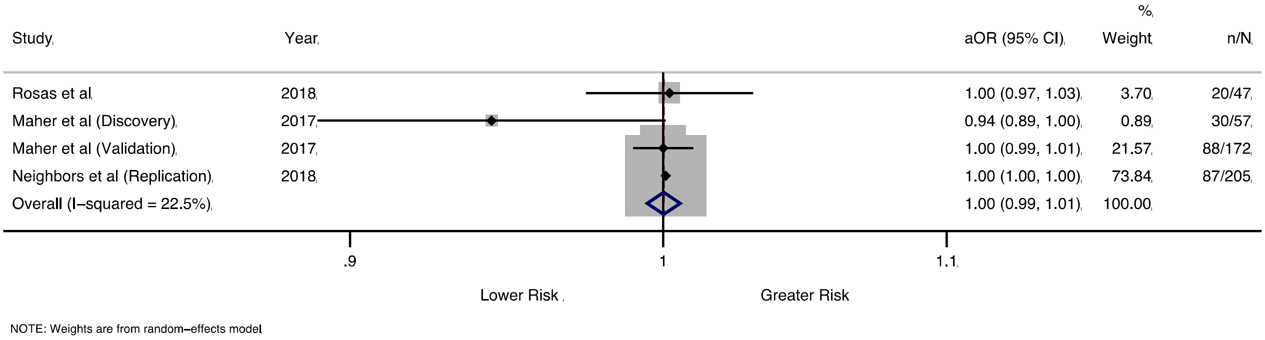

Supplementary Figure S8 – Pooled odds ratios with 95% confidence intervals for risk of disease progression, per percent relative increase in baseline MMP-7 to three months. n denotes the number of progressors, and N represents the total number of participants included in the analysis per study.

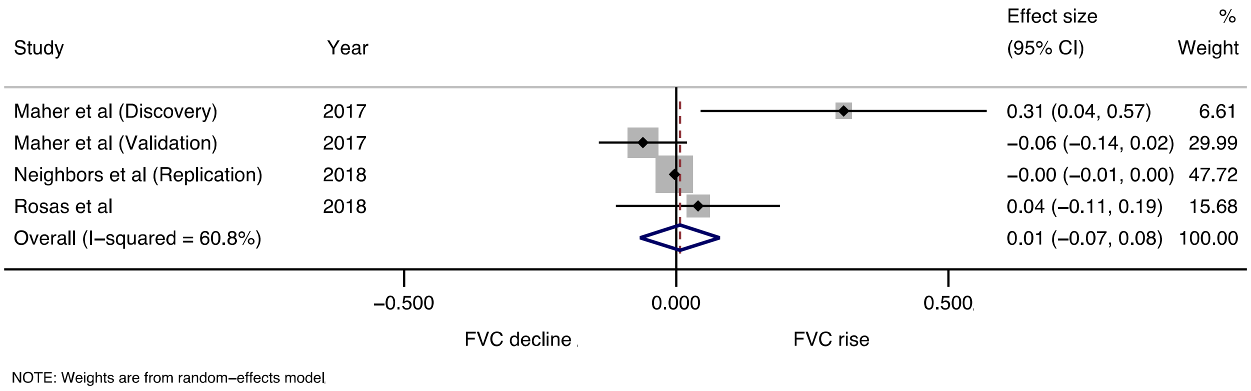

Supplementary Figure S9 – Pooled effect size with 95% confidence intervals for relative change in FVC at 12 months, per percent relative increase in baseline MMP-7 to three months.

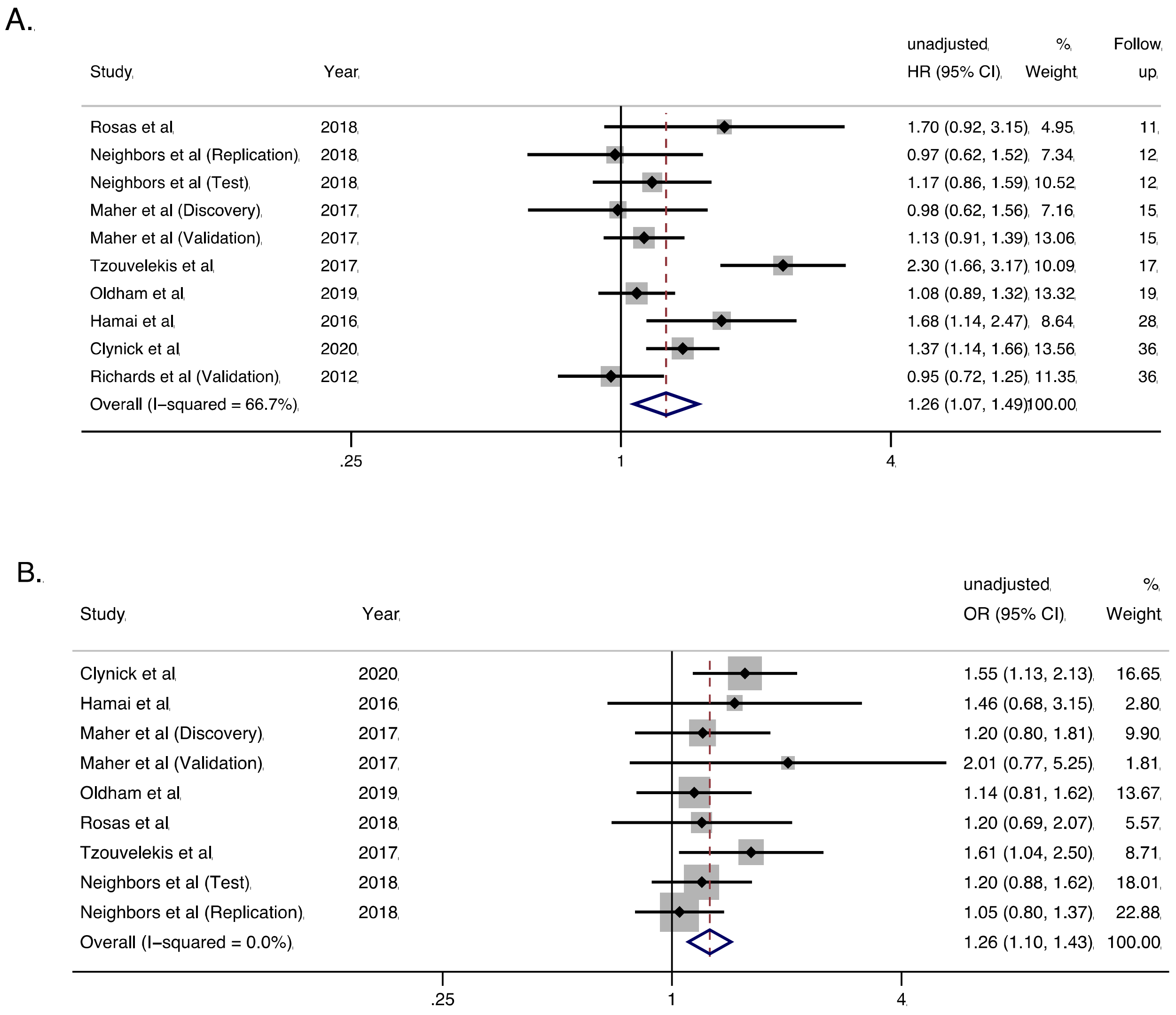

Supplementary Figure S10 – Unadjusted analyses including pooled estimates with 95% confidence intervals for association of baseline MMP-7 per standard deviation increase and A. Mortality, B. Disease progression.

Author and year of publication	Country of study	IPF Sample size	Study follow up, months	Age (years)	Sex – male (%)	Baseline FVC % predicted	Baseline DL_{CO} % predicted	Relevant Biomarkers evaluated	Relevant outcomes reported
Bauer, 2017[1]	multi-national	211 (BUILD-3[2])	NR	63.1 (8.9)	64	75.7 (10.7)	47.7 (10.7)	collagen synthesis peptides	Disease progression (FVC≥10% decline, DL_{CO} ≥ 15%, acute exacerbation or death) up to end of study, change in FVC at 4 months
Chien, 2014[3]	USA multi-national	69 (ARTEMIS[4])	24	66.2 (7)	75	69.8 (12.1)	42.1 (11.1)	LOXL2	Overall mortality, lung function decline at 24 months (FVC≥10% with DL_{CO} ≥ 5%, or DL_{CO} ≥ 15% with FVC ≥ 5%), disease progression (mortality, hospitalisation or lung function decline)
	USA multi-national	104 (GAP[5])		66.7 (8.9)	70	66.1 (17.7)	47.8 (18)		
Collard, 2010[6]	South Korea single centre	47 (AE-IPF)	NR	66 (8)	77	75 (18)	64 (20)	KL-6, SP-D	Overall mortality, acute exacerbation
		20 (without AE-IPF)		63 (7	80	84 (19)	74 (22)		
Doubkova, 2016[7]	Czech Republic single centre	18	NR	68.5 (49-79) [a]	56	68 (median)	52 (median)	SP-A, SP-D	Overall mortality, change in FVC
Gui, 2020[8]	China single centre	126	60	NR	75.4	70.1 (17)	50.5 (12.6)	KL-6, CXCL13	Overall mortality, change in FVC over 12 months
Hamai, 2016[9]	Japan single centre	65	31 (26.6-35.4) [b]	69.3 (8.6)	77	75.6 (21.9)	47.1 (15.8)	SP-A, SP-D, CCL-18, KL-6	5-year mortality
Hoyer, 2020[10]	Denmark multi-centre	184	36	NR	NR	NR	NR	PRO-C3, PRO-C6	Overall mortality, disease progression (FVC decline >10% and/or DL_{CO} decline >15% at any time)
Jiang, 2018[11]	China single centre	20 (85 ILD)	12	53.5 (10.5)	59 *	71.1 (17.7) *	49.4 (24.3) *	KL-6	Disease progression (FVC decline ≥ 10% or DL_{CO} decline ≥ 15%, or death) at 12 months
Jenkins, 2015[12]	UK multi-centre	55 (Discovery)	26 (1.6-35.2) [a]	68.5 (9.5)	78	75.9 (23.5)	44.4 (18.3)	ECM-neoepitopes	Overall mortality, disease progression at 12 months (all-cause mortality or >10% FVC decline)
		134 (Validation)	21.2 (0.8-36.2) [a]	70.7 (7.7)	79	78.1 (17.2)	42.1 (13.5)		
Kennedy, 2015[13]	Ireland single centre	13	6	72.6 (10.7)	77	83.3 (26.9)	39.1 (16.1)	SP-D	Change in FVC at 6 months
Kinder, 2009 [14]	USA single centre	82	36 (16-72) [b]	62 (10)	62	64 (18)	54 (16)	SP-A, SP-D	Death or transplantation at 1 year
Maher, 2017[15]	UK multi-centre	106 (Discovery)	36	70.8 (8.3)	78	79 (18.9)	43.3 (14.8)	SP-D, CA125, CA19-9, IGFBP-2, IL-8, ICAM-1	Overall mortality, disease progression at 12 months (all-cause mortality or FVC decline ≥ 10%)
		206 (Validation		72.5 (7.7)	76	81.4 (19.2)	49 (16.9)	SP-D, CA125, CA19-9	
Naik, 2012[16]	USA multi-centre	54 (COMET[17])	18.5	64.3 (8.2)	72	68.5 (15.8)	40. 8 (14.3)	Periostin	Disease progression at 48 weeks (death, acute exacerbation, transplantation, relative FVC decline ≥ 10% or DL_{CO} > 15%)

Neighbors, 2018[18]	multi-national	221 CAPACITY [19]	12	66.9 (7.4)	72	73.4 (13.4)	46.5 (9.4)	CCL-18, CXCL13, YKL-40, Periostin	At 12 months: Disease progression (FVC ≥10% absolute decline or death), change in FVC, death
		244 ASCEND [20]		67.7 (7.2)	77	68.3 (10.9)	43.9 (11.9)		
Ohshimo, 2014[21]	Germany single centre	64 (without AE-IPF)	36 (25.2)	70 (8)	73	68 (15)	44 (14)	KL-6, CCL-18	Acute exacerbation
		13 (with AE-IPF)		67 (5)	85	54 (17)	43 (10)		
Ohta, 2017[22]	Japan multi-centre	60	6.2 (5.8-8.5) [a]	69.2 (8.1)	92	85.8 (20.1)	59.7 (21.8)	Monomeric Periostin, Periostin, KL-6, SP-D	Change in FVC at 6-12 months
Okamoto, 2011[23]	Japan multi-centre	37	NR	66.3 (8.6)	84	80.2 (20)	NR	Periostin	Overall months
Organ, 2019[24]	UK multi-centre	145	34.5 (median)	71.7 (7.7)	81	79.8 (20.4)	48.2 (17.9)	ECM-neoepitopes, collagen synthesis peptides	Overall mortality, disease progression at 12 months (all-cause mortality or >10% FVC decline)
Papiris, 2018[25]	Greece single centre	23 (stable)	12	71 (69-74) [b]	82	72 (60-93) [b]	56 (38-65) [b]	IL-8	Overall mortality at 12 months
		18 (exacerbated)		68.5 (67-78) [b]	61	60 (44-64) [b]	35 (30-36) [b]		
Prasse, 2009[26]	Germany and Italy	72	24	67.2 (8.6)	NR	NR	NR	CCL-18	Overall mortality, change in FVC at 6 months, disease progression at 24 months (>10% FVC decline or death)
Raghu, 2018[27]	multi-national	154	12	67.9 (8.4)	64	71.5 (19.6)	40.9 (15.9)	SP-A, SP-D, CCL-18, KL-6, ICAM-1, Periostin, YKL-40	Disease progression at 52 weeks (FVC decrease ≥10% predicted or DL_{CO} decrease > 15% or lung transplantation or death)
Richards, 2012[28]	USA single centre	140 (Derivation)	22 (19)	67.2 (8.3)	72	62 (19.6)	44.8 (17.1)	IL-8, ICAM-1	Overall mortality, disease progression (FVC relative decline ≥ 10% within any 1 year of follow up)
		101 (Validation	17 (16)	68 (8.7)	66	60.8 (17)	45.4 (19)		
Vuga, 2014[29]	USA single centre	95	> 24	69 (9.7)	74	66 (19.5)	50 (19.5)	CXCL13	Overall mortality

Supplementary Table S1 – Methodological characteristics of all included non-MMP7 studies with baseline participant characteristics and outcome data. Age, baseline FVC and baseline DL_{CO} reported as mean (standard deviation) unless otherwise stated.
DL_{CO}, gas transfer for carbon monoxide; FVC, forced vital capacity; [a] = median and range; [b] = median and IQR
* = reported for all ILD

Study	Study participation	Study attrition	Prognostic factor	Outcome	Confounding	Statistical analysis and reporting
**IPD studies**						
Hamai, 2016	Moderate	Moderate	Low	Low	Low	Low
Maher, 2017	Low	Moderate	Low	Low	Low	Low
Navaratnam, 2014/Clynick, 2020	Low	Moderate	Low	Low	Low	Low
Neighbors, 2018	Low	Low	Low	Low	Low	Low
Oldham, 2019	Low	High	High	Low	High	Moderate
Raghu, 2018	Low	Low	Low	Low	Moderate	Low
Richards, 2012	Low	Low	Low	Low	Moderate	Low
Rosas, 2018	Low	Low	Low	Low	High	Moderate
Tzouvelekis, 2017	Low	Low	Low	Low	Low	Low
**Non-IPD studies**						
Bauer, 2017	Low	Low	Moderate	Low	High	Low
Chien, 2014	Low	Low	Low	Low	Moderate	Low
Collard, 2010	Low	Low	Low	Low	High	Low
Doubkova, 2016	Moderate	High	High	High	High	High
Gui, 2020	Low	Low	Low	Moderate	High	Low
Hoyer, 2020	High	High	High	Low	High	High
Jiang, 2018	Low	Low	Low	Low	High	Low
Jenkins, 2015	Low	Moderate	Low	Low	Low	Low
Kennedy, 2015	Moderate	Low	Low	Low	High	Moderate
Kinder, 2009	Low	Low	Low	Low	Low	Low
Naik, 2012	Low	Low	Low	Low	Low	Low
Ohshimo, 2014	Low	Low	Low	Low	Low	Low
Ohta, 2017	Low	High	Low	Low	High	Low
Okamoto, 2011	Low	High	Low	Low	Low	Moderate
Organ, 2019	Low	Moderate	Low	Low	Low	Low
Papiris, 2018	Low	Low	Low	Low	High	Moderate
Peljto, 2013	Low	Low	Moderate	Low	Low	Low
Prasse, 2009	Moderate	Low	Low	Low	Low	Low
Sokai, 2015	Low	Low	Low	Low	High	Low
Vuga, 2014	Moderate	High	Low	High	Low	Low

Supplementary Table S2 – Risk of bias assessment for included studies. The risk of bias across studies was rated as low, moderate or high risk in six categories using the QUIPs tool.

Baseline MMP-7								
Variables	Overall mortality (n=1492)		12-month mortality (n= 1492)		Disease progression (n= 1383)		Change in FVC percent predicted over 12 months (n=891)	
	R^2 (%)	P value	R^2 (%)	P value	R^2 (%)	P value	R^2 (%)	P value
Design (cohort vs. RCT)	0.00	0.747	0.00	0.388	0.00	0.159	0.00	0.988
Assay (ELISA vs. other)	18.45	0.088	25.4	0.075	100	0.013	0.00	0.235
Sample (Serum vs. plasma)	0.00	0.98	0.00	0.483	71.35	0.1875	0.00	0.502
IPF consensus (2011 vs. other)	0.00	0.983	0.00	0.87	100	0.05	N/A	N/A
Centre (single vs. multi)	9.05	0.1995	0.00	0.293	6.23	0.418	91.14	0.195
Publication type (peer reviewed)	0.00	0.922	0.00	0.893	47.51	0.212	0.00	0.659
**Change in MMP-7 over 3 months**								
Variables	Overall mortality (n=498)		12-month mortality (n=498)		Disease progression (n= 481)		Change in FVC percent predicted over 12 months (n= 481)	
	R^2 (%)	P value	R^2 (%)	P value	R^2 (%)	P value	R^2 (%)	P value
Design (cohort vs. RCT)	0.00	0.916	0.00	0.78	82.84	0.62	0.00	0.716
Assay (ELISA vs. other)	0.00	0.753	84.97	0.07	0.00	0.05	0.00	0.435
Sample (Serum vs. plasma)	0.00	0.56	0.00	0.557	19.2	0.662	0.00	0.716
IPF consensus (2011 vs. other)	N/A	N/A	N/A	N/A	N/A	N/A	N/A	N/A
Centre (single vs. multi)	N/A	N/A	N/A	N/A	N/A	N/A	N/A	N/A
Publication type (peer reviewed)	N/A	N/A	N/A	N/A	N/A	N/A	N/A	N/A

Supplementary Table S3 - Results of meta-regression for variables assessed separated by study outcomes. Sample sizes for each outcome shown (n). R^2 and p values from meta-regression shown where applicable.
N/A, not applicable.

Outcome	The GRADE domains	Ratings for quality of evidence
**Baseline MMP-7**		
Overall mortality (10 studies; 1492 participants)	Risk of bias	All studies included well-defined participants diagnosed according to international consensus guidelines. Exposure and outcomes were measured objectively and consistently for all participants. Covariates were adjusted for using IPD. Follow up was sufficiently long to enable outcomes to occur.
	Imprecision	Effect sizes in most studies favour MMP-7 as a marker of mortality.
	Inconsistency	Substantial heterogeneity not explained by variability in the factors assessed
	Indirectness	No serious indirectness. All patients had IPF according to consensus criteria and were untreated. MMP-7 was measured at baseline in all studies, and overall mortality measured from IPD.
	Publication bias	No publication bias as indicated by funnel plots and Egger's tests
	Certainty of evidence	Moderate certainty of evidence
12-month mortality (10 studies; 1492 participants)	Risk of bias	All studies included well-defined participants diagnosed according to international consensus guidelines. Exposure and outcomes were measured objectively and consistently for all participants. Covariates were adjusted for using IPD. Follow up was sufficiently long to enable outcomes to occur.
	Imprecision	Imprecision present with wide confidence interval of 0.99-1.78.
	Inconsistency	Substantial heterogeneity not explained by variability in the factors assessed
	Indirectness	No serious indirectness. All patients had IPF according to consensus criteria and were untreated. MMP-7 was measured at baseline in all studies, and 12-month mortality measured from IPD.
	Publication bias	No publication bias as indicated by funnel plots and Egger's tests

	Certainty of evidence	Moderate certainty of evidence
Disease progression (10 studies; 1383 participants)	Risk of bias	All studies included well-defined participants diagnosed according to international consensus guidelines. Exposure was measured objectively and consistently for all participants. Disease progression definition was standardised. Covariates were adjusted for using IPD. Follow up was sufficiently long to enable outcomes to occur.
	Imprecision	Effect sizes consistently favour MMP-7 as a prognostic marker, although confidence intervals commonly cross 1. Overall estimate has appropriately narrow confidence interval supporting MMP-7 as a biomarker of disease progression.
	Inconsistency	No heterogeneity demonstrated.
	Indirectness	No serious indirectness. All patients had IPF according to consensus criteria and were untreated. MMP-7 was measured at baseline in all studies, and disease progression standardised using IPD.
	Publication bias	No publication bias as indicated by funnel plots and Egger's tests
	Certainty of evidence	High certainty of evidence.
Change in FVC at 12 months (8 studies; 891 participants)	Risk of bias	All studies included well-defined participants diagnosed according to international consensus guidelines. Exposure was measured objectively and consistently for all participants. Change in FVC was measured objectively and consistently for all participants. Covariates were adjusted for using IPD. Follow up was sufficiently long to enable outcomes to occur.
	Imprecision	The majority of the studies show MMP-7 to result in a negative change in FVC at 12 months, although confidence intervals cross 0 in all individual studies. Overall confidence interval does not cross 0.
	Inconsistency	No evidence of heterogeneity
	Indirectness	No serious indirectness. All patients had IPF according to consensus criteria and were untreated. MMP-7 was measured at baseline in all studies and change in FVC standardised using IPD.
	Publication bias	No obvious funnel plot asymmetry, although unable to assess statistically due to small number of studies
	Certainty of evidence	High certainty of evidence.

**Three-month MMP-7 change**		
Overall mortality (4 studies; 498 participants)	Risk of bias	All studies included well-defined participants diagnosed according to international consensus guidelines. Exposure and outcomes were measured objectively and consistently for all participants. Covariates were adjusted for using IPD. Follow up was sufficiently long to enable outcomes to occur.
	Imprecision	Wide confidence intervals in individual studies but narrow confidence interval for overall effect size (no effect)
	Inconsistency	Substantial heterogeneity not explained by variability in the factors assessed
	Indirectness	No serious indirectness. All patients had IPF according to consensus criteria and were untreated. MMP-7 was measured at baseline in all studies, and overall mortality measured from IPD.
	Publication bias	No obvious funnel plot asymmetry, although unable to assess statistically due to small number of studies
	Certainty of evidence	Moderate certainty of evidence
12-month mortality (4 studies; 498 participants)	Risk of bias	All studies included well-defined participants diagnosed according to international consensus guidelines. Exposure and outcomes were measured objectively and consistently for all participants. Covariates were adjusted for using IPD. Follow up was sufficiently long to enable outcomes to occur.
	Imprecision	Wide confidence interval in individual studies but narrow confidence interval for overall effect size (no effect)
	Inconsistency	Heterogeneity not explained by variability in the factors assessed
	Indirectness	No serious indirectness. All patients had IPF according to consensus criteria and were untreated. MMP-7 was measured at baseline in all studies, and 12-month mortality measured from IPD.
	Publication bias	No obvious funnel plot asymmetry, although unable to assess statistically due to small number of studies
	Certainty of evidence	Moderate certainty of evidence

Disease progression (4 studies; 481 participants)	Risk of bias	All studies included well-defined participants diagnosed according to international consensus guidelines. Exposure and outcomes were measured objectively and consistently for all participants. Covariates were adjusted for using IPD. Follow up was sufficiently long to enable outcomes to occur.
	Imprecision	Wide confidence interval in individual studies but narrow confidence interval for overall effect size (no effect)
	Inconsistency	No significant heterogeneity
	Indirectness	No serious indirectness. All patients had IPF according to consensus criteria and were untreated. MMP-7 was measured at baseline in all studies, and overall mortality measured from IPD.
	Publication bias	No obvious funnel plot asymmetry, although unable to assess statistically due to small number of studies
	Certainty of evidence	High certainty of evidence
Change in FVC at 12 months (4 studies; 481 participants)	Risk of bias	All studies included well-defined participants diagnosed according to international consensus guidelines. Exposure was measured objectively and consistently for all participants. Change in FVC was measured objectively and consistently for all participants. Covariates were adjusted for using IPD. Follow up was sufficiently long to enable outcomes to occur.
	Imprecision	Wide confidence interval in individual studies but narrow confidence interval for overall effect size (no effect)
	Inconsistency	Inconsistency present across results from studies
	Indirectness	No serious indirectness. All patients had IPF according to consensus criteria and were untreated. MMP-7 was measured at baseline in all studies and change in FVC standardised using IPD.
	Publication bias	No obvious funnel plot asymmetry, although unable to assess statistically due to small number of studies
	Certainty of evidence	Moderate certainty of evidence.

Supplementary Table S4 – GRADE (Grading of Recommendations, Assessment, Development and Evaluations) approach to rate the quality of evidence for the prognostic factor MMP-7

Author (year)	Sample size	Follow up (months)	Effect size (Variance)	Level of adjustment	Effect size reported for
**MMP-7 (IPD unavailable)**					
Sokai (2015)	57	15	Not significant (NR)	NR	NR
Peljto (2013)	438	19	2.18 (95% CI 1.1-4.32)	b,d,e,h	bio > or < 5.7ng/mL
**SP-A**					
Kinder (2009)	82	36	HR 3.27 (95% CI 1.49-7.17)	a,b,c,d,e,g	per bio SD
Doubkova (2016)	18	NR	Not significant (NR)	x	bio > or < median (98.1ng/mL)
Hamai (2016)	65	31	HR 1.01 (95% CI 0.99-1.02)	x	continuous
**SP-D**					
Kinder (2009)	82	36	HR 2.04 (95% CI 0.99-4.22)	a,b,c,d,e,g	per bio SD
Collard (2010)	67	NR	OR 1.23 (95% CI 0.36-4.21)	"Bivariate" - NR	log change in bio
Doubkova (2016)	18	NR	Not significant (NR)	x	bio > or < median (623.1ng/mL)
Hamai (2016)	65	31	HR 1.00 (95% CI 0.99-1.002)	x	continuous
Maher (2017) - *Validation*	206	36	HR 2.72 (95% CI 1.65-4.48)	x	bio > or < 38.7ng/mL
**CCL-18**					
Prasse (2009)	72	24	HR 7.98 (95% CI 2.49-25.51)	a,b,c,d,e	bio > or < 150ng/mL
Hamai (2016)	65	31	HR 1.007 (95% CI 0.99-1.01)	X	continuous
Neighbors (2018) – *Test*	123	12	OR 4.4 (95% CI 1.13-17.15)	x	bio ≥ or < median
Neighbors (2018) – *Replication*	237	12	OR 3.37 (95% CI 1.17-9.67)	x	bio ≥ or < median
**CXCL-13**					
Guo (2020)	126	60	HR 1.03 (95% CI 1.02-1.06)	a	bio > or < 62pg/mL

Vuga (2014)	95	>24	HR 14.9 (95% CI 1.1-197.2)	a,b,d,e	bio > or < highest quartile
Neighbors (2018) – *Test*	123	12	OR 2.95 (95% CI 0.76-11.46)	x	bio ≥ or < median
Neighbors (2018) – *Replication*	237	12	OR 6.17 (95% CI 1.75-21.8)	x	bio ≥ or < median
**KL-6**					
Collard (2010)	67	NR	OR 0.41 (95% CI 0.06-2.93)	"Bivariate" - NR	bio log change
Hamai (2016)	65	31	HR 1.001 (95% CI 1.00-1.002)	a,b,c	continuous
Guo (2020)	126	60	HR 1.83 (95% CI 1.01-3.69)	a	bio > or < 800U/mL
**IL-8**					
Richards (2012) – *Derivation*	140	22	HR 2.4 (95% CI 1.2-4.79)	a,b,d	bio > or < 0.0029
Richards (2012) – *Validation*	101	17	HR 2.3 (95% CI 0.94-5.64)	a,b,d	bio > or < 0.0097
Papiris (2018)	41	12	OR 1.067 (95% CI 1.01-1.12)	x	per increase of 1pg/mL
**CA19-9**					
Maher (2017) – *Validation*	206	36	HR 2.95 (95% CI 1.82-4.78)	x	bio > or < 22 U/mL
**CA-125**					
Maher (2017) – *Validation*	206	36	HR 3.01 (95% CI 1.64-5.54)	x	bio > or < 12 U/mL
**LOXL2**					
Chien (2014) – *ARTEMIS*	69	24	HR 1.87 (95% CI 0.28-12.45)	d,e,f,h	bio > or ≤ 800pg/mL
Chien (2014) – *GAP*	104	24	HR 2.28 (95% CI 1.18-4.38)	b	bio > or ≤ 700pg/mL
**Periostin**					
Okamoto (2011)	77	36	Not significant (NR)	x	NR
Neighbors (2018) - *Test*	123	12	OR 3.05 (95% CI 0.79-11.88)	x	bio ≥ or < median
Neighbors (2018) – *Replication*	237	12	OR 1.91 (95% CI 0.72-5.05)	x	bio ≥ or < median
**YKL-40**					

Neighbors (2018) – *Test*	123	12	OR 1.77 (95% CI 0.53-5.92)	x	bio ≥ or < median
Neighbors (2018) – *Replication*	237	12	OR 2.7 (95% CI 0.94-7.75)	x	bio ≥ or < median
**ICAM-1**					
Richards (2012) - *Derivation*	140	22	HR 2.6 (95% CI 1.43-4.73)	a,b,d	bio > or < 202.5ng/mL
Richards (2012) – *Validation*	101	17	HR 2.8 (95% CI 1.36-5.76)	a,b,d	bio > or < 300ng/mL
**ECM neoepitopes**					
Jenkins (2015) –*Discovery* ***BGM***	55	26	HR 1.17 (95% CI 0.53-2.58)	x	two-fold increase in bio value
Jenkins (2015) –*Validation* ***BGM***	134	21	HR 1.34 (95% CI 0.92-1.97)	x	two-fold increase in bio value
Jenkins (2015) –*Discovery* ***C1M***	55	26	HR 1.21 (95% CI 0.66-2.22)	x	two-fold increase in bio value
Jenkins (2015) –*Validation* ***C1M***	134	21	HR 1.62 (95% CI 1.14-2.31)	x	two-fold increase in bio value
Jenkins (2015) –*Discovery* ***C3A***	55	26	HR 1.34 (95% CI 0.95-1.88)	x	two-fold increase in bio value
Jenkins (2015) –*Validation* ***C3A***	134	21	HR 1.91 (95% CI 1.06-3.46)	x	two-fold increase in bio value
Jenkins (2015) –*Discovery* ***C3M***	55	26	HR 2.18 (95% CI 0.95-5.00)	x	two-fold increase in bio value
Jenkins (2015) –*Validation* ***C3M***	134	21	HR 1.56 (95% CI 0.94-2.59)	x	two-fold increase in bio value
Jenkins (2015) –*Discovery* ***C5M***	55	26	HR 1.66 (95% CI 0.95-2.91)	x	two-fold increase in bio value
Jenkins (2015) –*Validation* ***C5M***	134	21	HR 1.07 (95% CI 0.66-1.72)	x	two-fold increase in bio value
Jenkins (2015) –*Discovery* ***C6M***	55	26	HR 1.49 (95% CI 0.86-2.56)	x	two-fold increase in bio value
Jenkins (2015) –*Validation* ***C6M***	134	21	HR 1.39 (95% CI 0.93-2.06)	x	two-fold increase in bio value
Jenkins (2015) –*Discovery* ***CRPM***	55	26	HR 3.74 (95% CI 1.46-9.58)	x	two-fold increase in bio value
Jenkins (2015) –*Validation* ***CRPM***	134	21	HR 1.87 (95% CI 0.98-3.56)	x	two-fold increase in bio value
Jenkins (2015) – *Discovery* ***ELM***	55	26	HR 0.96 (95% CI 0.48-1.92)	x	two-fold increase in bio value
Jenkins (2015) – *Discovery* ***ELM2***	55	26	HR 0.96 (95% CI 0.75-1.24)	x	two-fold increase in bio value

Jenkins (2015) – *Discovery* ***P3NP***	55	26	HR 1.48 (95% CI 0.67-3.27)	x	two-fold increase in bio value
Jenkins (2015) – *Discovery* ***VICM***	55	26	HR 1.11 (95% CI 0.83-1.49)	x	two-fold increase in bio value
**Collagen synthesis peptides**					
Organ (2019) **P1NP**	145	34	HR 0.81 (95% CI 0.6-1.11)	d,e	two-fold increase in bio value
Organ (2019) **PRO-C3**	145	34	HR 1.2 (95% CI 0.74-1.93)	d,e	two-fold increase in bio value
Hoyer (2020) **PRO-C3**	184	36	HR 2.32 (95% CI 1.33-4.04)	a	continuous
Organ (2019) **PRO-C6**	145	34	HR 1.11 (95% CI 0.57-2.16)	d,e	two-fold increase in bio value
Hoyer (2020) **PRO-C6**	184	36	HR 2.18 (95% CI 0.74-4.35)	a	continuous
Organ (2019) **P1NP:C1M**	145	34	HR 0.77 (95% CI 0.6-0.99)	d,e	two-fold increase in bio value
Organ (2019) **PRO-C3:C3M**	145	34	HR 1.17 (95% CI 0.77-1.79)	d,e	two-fold increase in bio value
Organ (2019) **PRO-C6:C6M**	145	34	HR 0.86 (95% CI 0.59-1.26)	d,e	two-fold increase in bio value
Hoyer (2020) **PRO-C6**	184	36	HR 1.8 (95% CI 0.74-4.35)	a	continuous

Supplementary Table S5 – Studies reporting mortality outcomes
x=no adjustments, a=age, b=gender, c=smoking, d=FVC e=DLCO, f= 6MWT, g=race, h=medication

bio, biomarker; HR, hazard ratio; IPD, individual participant data; NR, not reported; OR, odds ratio

Author (year)	Sample size	Follow up (months)	Effect size (Variance)	Level of adjustment	Effect size reported for
**SP-D**					
Maher (2017) - *Discovery*	106	36	HR 1.01 (95% CI 0.97-1.06)	x	rising vs stable bio over 3 months
Maher (2017) – *Validation*	206	36	HR 0.99 (95% CI 0.59-1.67)	a,b,c,d	rising vs stable bio over 3 months
**CA19-9**					
Maher (2017) - *Discovery*	106	36	HR 1.02 (95% CI 1.00-1.05)	X	rising vs stable bio over 3 months
Maher (2017) – *Validation*	206	36	HR 1.39 (95% CI 0.79-2.46)	a,b,c,d	rising vs stable bio over 3 months
**CA-125**					
Maher (2017) - *Discovery*	106	36	HR 1.77 (95% CI 1.39-2.26)	x	rising vs stable bio over 3 months
Maher (2017) – *Validation*	206	36	HR 2.39 (95% CI 1.4-4.08)	a,b,c,d	rising vs stable bio over 3 months
**ICAM-1**					
Maher (2017) - *Discovery*	106	36	HR 1.002 (95% CI 0.99-1.01)	x	rising vs stable bio over 3 months
**IGFBP-2**					
Maher (2017) - *Discovery*	106	36	HR 1.02 (95% CI 1.002-1.03)	x	rising vs stable bio over 3 months
**IL-8**					
Maher (2017) - *Discovery*	106	36	HR 1.02 (95% CI 0.98-1.07)	x	rising vs stable bio over 3 months
**ECM neoepitopes**					
Jenkins (2015) –*Validation* ***BGM***	134	21	HR 1.07 (95% CI 1.00-1.15)	a,c,d,e	rising vs stable bio over 3 months
Organ (2019) **BGM**	145	34	HR 1.41 (95% CI 0.8-2.47)	a,b,c	rising vs stable bio over 3 months
Jenkins (2015) –*Validation* ***C1M***	134	21	HR 1.01 (95% CI 1.00-1.02)	a,c,d,e	rising vs stable bio over 3 months
Organ (2019) **C1M**	145	34	HR 1.84 (95% CI 1.03-3.27)	a,b,c	rising vs stable bio over 3 months

Jenkins (2015) –*Validation* ***C3A***	134	21	HR 1.05 (95% CI 1.01-1.1)	a,c,d,e	rising vs stable bio over 3 months
Jenkins (2015) –*Validation* ***C3M***	134	21	HR 1.1 (95% CI 1.04-1.17)	a,c,d,e	rising vs stable bio over 3 months
Organ (2019) **C3M**	145	34	HR 2.44 (95% CI 1.39-4.31)	a,b,c	rising vs stable bio over 3 months
Jenkins (2015) –*Validation* ***C5M***	134	21	HR 1.00 (95% CI 1.00-1.00)	a,c,d,e	rising vs stable bio over 3 months
Jenkins (2015) –*Validation* ***C6M***	134	21	HR 1.04 (95% CI 1.01-1.08)	a,c,d,e	rising vs stable bio over 3 months
Organ (2019) **C6M**	145	34	HR 2.19 (95% CI 1.25-3.82)	a,b,c	rising vs stable bio over 3 months
Jenkins (2015) –*Validation* ***CRPM***	134	21	HR 1.33 (95% CI 1.1-1.6)	a,c,d,e	rising vs stable bio over 3 months
Organ (2019) **CRPM**	145	34	HR 2.13 (95% CI 1.21-3.75)	a,b,c	rising vs stable bio over 3 months
Jenkins 2015) – *Validation* ***VICM***	55	26	HR 1.01 (95% CI 0.99-1.03)	a,c,d,e	rising vs stable bio over 3 months
**Collagen synthesis peptides**					
Organ (2019) **P1NP**	145	34	HR 0.76 (95% CI 0.44-1.3)	a,b,c	rising vs stable bio over 3 months
Organ (2019) **PRO-C3**	145	34	HR 1.62 (95% CI 0.95-2.79)	a,b,c	rising vs stable bio over 3 months
Organ (2019) **PRO-C6**	145	34	HR 1.14 (95% CI 0.67-1.93)	a,b,c	rising vs stable bio over 3 months
Organ (2019) **P1NP:C1M**	145	34	HR 0.73 (95% CI 0.41-1.29)	a,b,c	rising ratio levels
Organ (2019) **PRO-C3:C3M**	145	34	HR 0.83 (95% CI 0.49-1.43)	a,b,c	rising ratio levels
Organ (2019) **PRO-C6:C6M**	145	34	HR 0.55 (95% CI 0.32-0.95)	a,b,c	rising ratio levels

Supplementary Table S6 – Studies reporting short term biomarkers change and their association with mortality

x=no adjustments, a=age, b=gender, c=smoking, d=FVC e=DLCO, f= 6MWT, g=race, h=medication
bio, biomarker; HR, hazard ratio.

Author (year)	Sample size	Timepoint of outcome (months)	Disease progression definition	Effect size (Variance)	Level of adjustment	Effect size reported for
**MMP-7 (IPD unavailable)**						
Sokai (2015)	57	6	FVC decline ≥10% or DL_{CO} ≥ 15% decline or respiratory failure or death	Not significant (NR)	NR	NR
Bauer (2017)	211	19	FVC decline ≥ 10% or DL_{CO} ≥ 15% decline or respiratory failure or death	HR 2.2 (95% CI 1.4-3.7)	NR	bio < or ≥ 3.8ng/mL
**SP-A**						
Raghu (2018)	130	12	FVC decrease ≥10% predicted or DL_{CO} decrease > 15% or lung transplantation or death	AUROC 0.61 (90% CI 0.52-0.7)	NR	NR
**SP-D**						
Collard (2010)	67	NR	Acute exacerbation	361ng/mL vs 294ng/mL (p=0.01)	x	median bio in event and non-event group
Maher (2017) *Discovery*	104	12	All-cause mortality or FVC decline ≥ 10%	GR 1.35 (95% CI 1.1-1.649)	x	bio level in progressive vs. stable group
Maher (2017) *Validation*	204	12	All-cause mortality or FVC decline ≥ 10%	GR 1.35 (95% CI 1.12-1.62)	x	bio level in progressive vs. stable group
Raghu (2018)	130	12	FVC decrease ≥10% predicted or DL_{CO} decrease > 15% or lung transplantation or death	AUROC 0.62 (90% CI 0.53-0.7)	NR	NR
**CCL-18**						
Prasse (2009)	67	24	FVC decline ≥ 10% prediced or death	OR 6.75 (95% CI 2.52-18.1)	x	bio < or > 150ng/mL
Ohshimo (2014)	77	36	Acute exacerbation	HR 2.92 (95% CI 0.76-11.4)	x	bio > or < 212ng/mL
Neighbors (2018) *Test*	123	12	FVC ≥10% absolute decline, 50m decline in 6MWT or death	HR 1.64 (95% CI 1.04-2.83)	x	'high' vs 'low' bio
Neighbors (2018) *Replication*	237	12	FVC ≥10% absolute decline, 50m decline in 6MWT or death	HR 1.32 (95% CI 0.76-2.13)	x	'high' vs 'low' bio
Raghu (2018)	130	12	FVC decrease ≥10% predicted or DL_{CO} decrease > 15% or lung transplantation or death	AUROC 0.62 (90% CI 0.54-0.71)	NR	bio > or < 150ng/mL

**CXCL-13**						
Neighbors (2018) *Test*	123	12	FVC ≥10% absolute decline, 50m decline in 6MWT or death	HR 1.23 (95% CI 0.89-1.69)	x	'high' vs 'low' bio
Neighbors (2018) *Replication*	237	12	FVC ≥10% absolute decline, 50m decline in 6MWT or death	Not significant (NR)	x	'high' vs 'low' bio
**KL-6**						
Collard (2010)	67	NR	Acute exacerbation	1791 U/mL vs 895 U/mL (p=0.003)	x	median bio in event and non-event group
Ohshimo (2014)	77	36	Acute exacerbation	HR 11.8 (95% CI 1.43-97.8)	a,b,c,h	bio > or < 1300U/mL
Jiang (2018)	20	12	FVC decline ≥ 10% or DL_{CO} decline ≥ 15%, or death	OR 1.00 (95% CI 1.00-1.00)	x	continuous bio
Raghu (2018)	130	12	FVC decrease ≥10% predicted or DL_{CO} decrease > 15% or lung transplantation or death	AUROC 0.6 (90% CI 0.51-0.68)	NR	NR
**IL-8**						
Richards (2012) *Derivation*	140	12	FVC relative decline ≥ 10%	HR 2.00 (95% CI 1.22-3.28)	a,b,d	bio > or < 0.0092ng/mL
Richards (2012) *Validation*	101	12	FVC relative decline ≥ 10%	HR 1.2 (95% CI 0.5-2.85)	a,b,d	bio > or < 0.0092ng/mL
Maher (2017) *Discovery*	104	12	All-cause mortality or FVC decline ≥ 10%	GR 1.51 (95% CI 1.12-2.023)	x	bio level in progressive vs. stable group
**CA19-9**						
Maher (2017) *Discovery*	104	12	All-cause mortality or FVC decline ≥ 10%	GR 3.12 (95% CI 1.7-5.7)	x	bio level in progressive vs. stable group
Maher (2017) *Validation*	204	12	All-cause mortality or FVC decline ≥ 10%	GR 2.42 (95% CI 1.6-3.65)	x	bio level in progressive vs. stable group
**CA125**						
Maher (2017) *Discovery*	104	12	All-cause mortality or FVC decline ≥ 10%	Not significant (NR)	x	bio level in progressive vs. stable group
Maher (2017) *Validation*	204	12	All-cause mortality or FVC decline ≥ 10%	GR 1.26 (95% CI 1.05-1.51)	x	bio level in progressive vs. stable group

**LOXL2**						
Chien (2014) *ARTEMIS*	69	24	Mortality, hospitalisation or lung function decline (FVC≥10% & DL_{CO}≥5%, or DL_{CO} ≥ 15% and FVC≥5%)	HR 5.41 (95% CI 1.65-17.73)	d,e,f,h	bio > or ≤ 800pg/mL
Chien (2014) *GAP*	70	24	Mortality, hospitalisation or lung function decline (FVC≥10% & DL_{CO}≥5%, or DL_{CO} ≥ 15% and FVC≥5%)	HR 1.78 (95% CI 1.01-3.11)	x	bio > or ≤ 700pg/mL
**Periostin**						
Naik (2012)	50	11	Death, acute exacerbation, transplantation, relative FVC decline ≥ 10% or DL_{CO} > 15%	HR 1.47 (95% CI 1.03-2.1)	a,b,c,d,e	per bio SD
Neighbors (2018) *Test*	123	12	FVC ≥10% absolute decline, 50m decline in 6MWT or death	HR 2.08 (95% CI 1.24-3.47)	x	'high' vs 'low' bio
Neighbors (2018) *Replication*	237	12	FVC ≥10% absolute decline, 50m decline in 6MWT or death	HR 1.75 (95% CI 0.87-2.84)	x	'high' vs 'low' bio
Raghu (2018)	130	12	FVC decrease ≥10% predicted or DL_{CO} decrease > 15% or lung transplantation or death	AUROC 0.6 (90% CI 0.51-0.69)	NR	NR
**YKL-40**						
Neighbors (2018) *Test*	123	12	FVC ≥10% absolute decline, 50m decline in 6MWT or death	HR 1.39 (95% CI 0.79-2.41)	x	'high' vs 'low' bio
Neighbors (2018) *Replication*	237	12	FVC ≥10% absolute decline, 50m decline in 6MWT or death	Not significant (NR)	x	'high' vs 'low' bio
Raghu (2018)	130	12	FVC decrease ≥10% predicted or DL_{CO} decrease > 15% or lung transplantation or death	AUROC 0.58 (90% CI 0.49-0.67)	NR	NR
ICAM-1						
Richards (2012) *Derivation*	140	12	FVC relative decline ≥ 10%	HR 1.6 (95% CI 1.00-2.56)	a,b,d	bio > or < 202.5ng/mL
Richards (2012) *Validation*	101	12	FVC relative decline ≥ 10%	HR 2.2 (95% CI 1.21-4.01)	a,b,d	bio > or < 262ng/mL
Maher (2017) *Discovery*	104	12	All-cause mortality or FVC decline ≥ 10%	GR 1.29 (95% CI 1.02-1.65)	x	bio level in progressive vs. stable group
Raghu 2018	130	12	FVC decrease ≥10% predicted or DL_{CO} decrease > 15% or lung transplantation or death	AUROC 0.65 (90% CI 0.56-0.73)	NR	NR
**ECM neoepitopes**						

Jenkins (2015) *D+V cohort* **BGM**	186	12	All-cause mortality or FVC decline ≥ 10%	Not significant (NR)	x	bio level in progressive vs. stable group
Jenkins (2015) *D+V cohort* **C1M**	186	12	All-cause mortality or FVC decline ≥ 10%	Not significant (NR)	x	bio level in progressive vs. stable group
Jenkins (2015) *D+V cohort* **C3M**	186	12	All-cause mortality or FVC decline ≥ 10%	P=0.011 (NR)	x	bio level in progressive vs. stable group
Jenkins (2015) *D+V cohort* **C5M**	186	12	All-cause mortality or FVC decline ≥ 10%	Not significant (NR)	x	bio level in progressive vs. stable group
Jenkins (2015) *D+V cohort* **C6M**	186	12	All-cause mortality or FVC decline ≥ 10%	P=0.013 (NR	x	bio level in progressive vs. stable group
Jenkins (2015) *D+V cohort* **CRPM**	186	12	All-cause mortality or FVC decline ≥ 10%	P=0.014 (NR)	x	bio level in progressive vs. stable group
Jenkins (2015) *D+V cohort* **VICM**	186	12	All-cause mortality or FVC decline ≥ 10%	P=0.033 (NR)	x	bio level in progressive vs. stable group
Jenkins (2015) *D+V cohort* **C3A**	186	12	All-cause mortality or FVC decline ≥ 10%	P=0.003 (NR)	x	bio level in progressive vs. stable group
Jenkins (2015) *Discovery only* **P3NP**	186	12	All-cause mortality or FVC decline ≥ 10%	P=0.63 (NR)	x	bio level in progressive vs. stable group
Jenkins (2015) *Discovery only* **ELM**	186	12	All-cause mortality or FVC decline ≥ 10%	P=0.55 (NR)	x	bio level in progressive vs. stable group
Jenkins (2015) *Discovery only* **ELM2**	186	12	All-cause mortality or FVC decline ≥ 10%	P=0.42 (NR)	x	bio level in progressive vs. stable group
Hoyer (2020) **PROC3**	184	6	All-cause mortality or FVC decline ≥ 10%	P=0.005 (NR)	NR	NR
Hoyer (2020) **PROC6**	184	6	All-cause mortality or FVC decline ≥ 10%	P=0.031 (NR)	NR	NR

Supplementary Table S7 – Studies reporting disease progression outcomes including definition of disease progression outcome used and effect sizes reported.

x=no adjustments, a=age, b=gender, c=smoking, d=FVC e=DL_{CO}, f= 6MWT, g=race, h=medication, NR=not reported

bio, biomarker; AUROC; area under the receiver operating characteristics; $DL_{CO,}$ gas transfer for carbon monoxide; FVC, forced vital capacity; GR, group ratio; HR, hazard ratio; IPD, individual participant data; NR, not reported; OR, odds ratio; 6MWT, 6-minute walk test;

Author (year)	Sample size	FVC change measured at (months)	Effect size (Variance)	Level of adjustment	Effect size reported for
**MMP-7 (IPD unavailable)**					
Bauer (2017)	195	4	p=0.004 (NR)	x	baseline bio correlation with %pred FVC change
**SP-A**					
Doubkova (2016)	18	NR	155.8 ng/mL vs 87.15 ng/mL; p=0.01	x	baseline bio in PFT "improvement" vs "stabilisation"
**SP-D**					
Doubkova (2016)	18	NR	861.4ng/mL vs. 802.8ng/mL; p=0.76	x	baseline bio in PFT "improvement" vs "stabilisation"
Kennedy (2015)	13	6	r= -0.64 (95% CI -0.89 to -0.08)	x	baseline bio correlation with %pred FVC change
Ohta (2017)	60	6-12	r= 0.09 (p>0.05)	x	baseline bio correlation with %pred FVC change
**CCL-18**					
Neighbors (2018) – *Test*	123	12	-3.1% (p=0.03)	x	%pred FVC change in baseline bio ≥ or < median (411.5ng/mL)
Neighbors (2018) – *Replication*	237	12	-3.6% (p=0.004)	x	%pred FVC change in baseline bio ≥ or < median (458.6ng/mL)
Prasse (2009)	67	6	r=0.54 (p<0.0001)	x	baseline bio correlation with %pred FVC change
**CXCL-13**					
Guo (2020)	126	12	r= 0.56 (p<0.001)	x	baseline bio correlation with %pred FVC change
Neighbors (2018) – *Test*	123	12	-3.2% (p=0.06)	x	%pred FVC change in baseline bio ≥ or < median (87.9ng/mL)
Neighbors (2018) – *Replication*	237	12	-3.7% (p=0.05)	x	%pred FVC change in baseline bio ≥ or < median (88.7ng/mL)
**KL-6**					
Guo (2020)	126	12	r= 0.71 (p<0.001)	x	baseline bio correlation with %pred FVC change
Ohta (2017)	60	6-12	r= 0.09 (p>0.05)	x	baseline bio correlation with %pred FVC change
Okamoto (2011)	26	6	Not significant (NR)	x	baseline bio correlation with %pred FVC change

**Periostin**					
Neighbors (2018) – *Test*	123	12	-3.6% (p<0.001)	x	%pred FVC change in baseline bio ≥ or < median (67.8ng/mL)
Neighbors (2018) – *Replication*	237	12	-2.5% (p=0.19)	x	%pred FVC change in baseline bio ≥ or < median (65.4ng/mL)
Ohta (2017)	60	6-12	r= -0.43 (p<0.01)	x	baseline bio correlation with %pred FVC change
Okamoto (2011)	26	6	r= -0.50 (p<0.01)	x	baseline bio correlation with %pred FVC change
**YKL-40**					
Neighbors (2018) – *Test*	123	12	-2.4% (p=0.04)	x	%pred FVC change in baseline bio ≥ or < median (100.3ng/mL)
Neighbors (2018) – *Replication*	237	12	-1.5% (p=0.70)	x	%pred FVC change in baseline bio ≥ or < median (109.5ng/mL)

Supplementary Table S8 – Studies reporting association with baseline biomarkers and change in forced vital capacity (FVC).
bio, biomarker; x = no adjustments

IPD, individual participant data.

Author (year)	Sample size	Timepoint of outcome (months)	Disease progression definition	Effect size (Variance)	Level of adjustment	Effect size reported for
**MMP-7 (IPD unavailable)**						
Bauer et al (2017)	211	"Study period"	FVC ≥10% decline, DL_{CO} ≥ 15%, acute exacerbation or death	OR 1.9 (95% CI 1.2-3.0)	NR	Two-fold change in bio over 4 months
**SP-D**						
Maher et al (2017) *Discovery*	106	12	All-cause mortality or FVC decline ≥ 10%	p=0.029	x	rising vs stable bio over 3 months
Maher et al (2017) *Validation*	206	12	All-cause mortality or FVC decline ≥ 10%	Not significant (NR)	x	rising vs stable bio over 3 months
**CXCL-13**						
Vuga et al (2014)	95	>24	Respiratory failure	HR 7.2 (95% CI 1.3-40.0)	x	bio "increase greatest vs. less increased" (time not specified)
**CA19-9**						
Maher et al (2017) *Discovery*	106	12	All-cause mortality or FVC decline ≥ 10%	p<0.001	x	rising vs stable bio over 3 months
Maher et al (2017) *Validation*	206	12	All-cause mortality or FVC decline ≥ 10%	Not significant (NR)	x	rising vs stable bio over 3 months
**CA125**						
Maher et al (2017) *Discovery*	106	12	All-cause mortality or FVC decline ≥ 10%	p=0.041	x	rising vs stable bio over 3 months
Maher et al (2017) *Validation*	206	12	All-cause mortality or FVC decline ≥ 10%	p=0.0028	x	rising vs stable bio over 3 months
**KL-6**						
Jiang et al (2018)	20	12	FVC decline ≥ 10%, DL_{CO} decline ≥ 15% or death	OR 3.61 (95% CI 1.05-6.22)	a,b,c,d,e	Change in KL-6 (not otherwise specified)

Supplementary Table S9 – Studies reporting short term biomarkers change and their association with disease progression

x=no adjustments, a=age, b=gender, c=smoking, d=FVC e=DL_{CO}, f= 6MWT, g=race, h=medication, NR=not reported
bio, biomarker; DL_{CO}, gas transfer for carbon monoxide; FVC, forced vital capacity; GR, group ratio; HR, hazard ratio; IPD, individual participant data; NR, not reported; OR, odds ratio

References

1. Bauer Y, White ES, de Bernard S, et al. MMP-7 is a predictive biomarker of disease progression in patients with idiopathic pulmonary fibrosis. *ERJ Open Research* 2017;3 (1) (no pagination)(00074-2016)
2. King TE, Jr., Brown KK, Raghu G, et al. BUILD-3: a randomized, controlled trial of bosentan in idiopathic pulmonary fibrosis. *American Journal of Respiratory & Critical Care Medicine* 2011;184(1):92-9.
3. Chien JW, Richards TJ, Gibson KF, et al. Serum lysyl oxidase-like 2 levels and idiopathic pulmonary fibrosis disease progression. *European Respiratory Journal* 2014;43(5):1430-8.
4. Raghu G, Behr J, Brown KK, et al. Treatment of idiopathic pulmonary fibrosis with ambrisentan: a parallel, randomized trial. *Ann Intern Med* 2013;158(9):641-9. doi: 10.7326/0003-4819-158-9-201305070-00003 [published Online First: 2013/05/08]
5. Genomic and Proteomic Analysis of Disease Progression in Idiopathic Pulmonary Fibrosis (IPF) (GAP) [Available from: https://clinicaltrials.gov/ct2/show/NCT00373841.
6. Collard HR, Calfee CS, Wolters PJ, et al. Plasma biomarker profiles in acute exacerbation of idiopathic pulmonary fibrosis. *American Journal of Physiology - Lung Cellular and Molecular Physiology* 2010;299(1):L3-L7.
7. Doubkova M, Karpisek M, Mazoch J, et al. Prognostic significance of surfactant protein A, surfactant protein D, Clara cell protein 16, S100 protein, trefoil factor 3, and prostatic secretory protein 94 in idiopathic pulmonary fibrosis, sarcoidosis, and chronic pulmonary obstructive disease. *Sarcoidosis Vasculitis & Diffuse Lung Diseases* 2016;33(3):224-34.
8. Guo L, Liu F, Jiang C, et al. Clinical Research on Prognostic Evaluation of Subjects With IPF by Peripheral Blood Biomarkers, Quantitative Imaging Characteristics and Pulmonary Function Parameters. *Archivos de Bronconeumologia* 2020;56(6):365-72.
9. Hamai K, Iwamoto H, Ishikawa N, et al. Comparative Study of Circulating MMP-7, CCL18, KL-6, SP-A, and SP-D as Disease Markers of Idiopathic Pulmonary Fibrosis. *Dis Markers* 2016;2016:4759040. doi: 10.1155/2016/4759040 [published Online First: 2016/06/14]
10. Hoyer N, Jessen H, Rønnow S, et al. Biomarkers of collagen formation are predictive of IPF progression and death. *European Respiratory Journal* 2020;56(suppl 64):5186. doi: 10.1183/13993003.congress-2020.5186
11. Jiang Y, Luo Q, Han Q, et al. Sequential changes of serum KL-6 predict the progression of interstitial lung disease. *Journal of Thoracic Disease* 2018;10(8):4705-14.
12. Jenkins RG, Simpson JK, Saini G, et al. Longitudinal change in collagen degradation biomarkers in idiopathic pulmonary fibrosis: An analysis from the prospective, multicentre PROFILE study. *The Lancet Respiratory Medicine* 2015;3(6):462-72.
13. Kennedy B, Branagan P, Moloney F, et al. Biomarkers to identify ILD and predict lung function decline in scleroderma lung disease or idiopathic pulmonary fibrosis. *Sarcoidosis Vasculitis and Diffuse Lung Diseases* 2015;32(3):228-36.
14. Kinder BW, Brown KK, McCormack FX, et al. Serum surfactant protein-A is a strong predictor of early mortality in idiopathic pulmonary fibrosis. *Chest* 2009;135(6):1557-63.

15. Maher TM, Oballa E, Simpson JK, et al. An epithelial biomarker signature for idiopathic pulmonary fibrosis: an analysis from the multicentre PROFILE cohort study. *The Lancet Respiratory Medicine* 2017;5(12):946-55.
16. Naik PK, Bozyk PD, Bentley JK, et al. Periostin promotes fibrosis and predicts progression in patients with idiopathic pulmonary fibrosis. *American Journal of Physiology - Lung Cellular & Molecular Physiology* 2012;303(12):L1046-56.
17. Han MK, Zhou Y, Murray S, et al. Lung microbiome and disease progression in idiopathic pulmonary fibrosis: An analysis of the COMET study. *The Lancet Respiratory Medicine* 2014;2(7):448-56.
18. Neighbors M, Cabanski CR, Ramalingam TR, et al. Prognostic and predictive biomarkers for patients with idiopathic pulmonary fibrosis treated with pirfenidone: post-hoc assessment of the CAPACITY and ASCEND trials. *The Lancet Respiratory Medicine* 2018;6(8):615-26.
19. Noble PW, Albera C, Bradford WZ, et al. Pirfenidone in patients with idiopathic pulmonary fibrosis (CAPACITY): two randomised trials. *Lancet (London, England)* 2011;377(9779):1760-9. doi: 10.1016/s0140-6736(11)60405-4 [published Online First: 2011/05/17]
20. King TE, Jr., Bradford WZ, Castro-Bernardini S, et al. A phase 3 trial of pirfenidone in patients with idiopathic pulmonary fibrosis.[Erratum appears in N Engl J Med. 2014 Sep 18;371(12):1172]. *New England Journal of Medicine* 2014;370(22):2083-92.
21. Ohshimo S, Ishikawa N, Horimasu Y, et al. Baseline KL-6 predicts increased risk for acute exacerbation of idiopathic pulmonary fibrosis. *Respiratory Medicine* 2014;108(7):1031-39.
22. Ohta S, Okamoto M, Fujimoto K, et al. The usefulness of monomeric periostin as a biomarker for idiopathic pulmonary fibrosis. *PLoS ONE [Electronic Resource]* 2017;12(3):e0174547.
23. Okamoto M, Hoshino T, Kitasato Y, et al. Periostin, a matrix protein, is a novel biomarker for idiopathic interstitial pneumonias. *The European respiratory journal* 2011;37(5):1119-27. doi: 10.1183/09031936.00059810 [published Online First: 2010/12/24]
24. Organ LA, Duggan A-MR, Oballa E, et al. Biomarkers of collagen synthesis predict progression in the PROFILE idiopathic pulmonary fibrosis cohort. *Respiratory Research* 2019;20(1):148. doi: 10.1186/s12931-019-1118-7
25. Papiris SA, Tomos IP, Karakatsani A, et al. High levels of IL-6 and IL-8 characterize early-on idiopathic pulmonary fibrosis acute exacerbations. *Cytokine* 2018;102:168-72.
26. Prasse A, Probst C, Bargagli E, et al. Serum CC-chemokine ligand 18 concentration predicts outcome in idiopathic pulmonary fibrosis. *American Journal of Respiratory and Critical Care Medicine* 2009;179(8):717-23.
27. Raghu G, Richeldi L, Jagerschmidt A, et al. Idiopathic Pulmonary Fibrosis: Prospective, Case-Controlled Study of Natural History and Circulating Biomarkers. *Chest* 2018;154(6):1359-70.
28. Richards TJ, Kaminski N, Baribaud F, et al. Peripheral blood proteins predict mortality in idiopathic pulmonary fibrosis.[Erratum appears in Am J Respir Crit Care Med. 2012 Feb 15;185(4):464]. *American Journal of Respiratory & Critical Care Medicine* 2012;185(1):67-76.

29. Vuga LJ, Tedrow JR, Pandit KV, et al. C-X-C motif chemokine 13 (CXCL13) is a prognostic biomarker of idiopathic pulmonary fibrosis. *American Journal of Respiratory & Critical Care Medicine* 2014;189(8):966-74.
