# Supplementary material for: A systematic review of blood biomarkers with individual participant data meta-analysis of matrix metalloproteinase-7 in idiopathic pulmonary fibrosis
Source: Eur Respir J. 2022 Apr 28;59(4):2101612. doi: 10.1183/13993003.01612-2021 (PMC9202487; doi:10.1183/13993003.01612-2021)
Supplement: Supplementary file 2 [file ERJ-01612-2021.Shareable.pdf]

SHAREABLE PDF

A systematic review of blood biomarkers with individual participant data meta-analysis of matrix metalloproteinase-7 in idiopathic pulmonary fibrosis

Fasihul A. Khan[1,2], Iain Stewart[1,2,3], Gauri Saini[1], Karen A. Robinson[4] and R. Gisli Jenkins[1,2,3]

[1]Division of Respiratory Medicine, School of Medicine, University of Nottingham, Nottingham, UK. [2]Nottingham Biomedical Research Centre, National Institute for Health Research, Nottingham, UK. [3]Margaret Turner Warwick Centre for Fibrosing Lung Disease, National Health and Lung Institute, Imperial College London, London, UK. [4]Epidemiology and Health Policy and Management, Johns Hopkins University, Baltimore, MD, USA.

Corresponding author: Fasihul A. Khan (fasihul.khan@nottingham.ac.uk)

Check for updates

Shareable abstract (@ERSpublications)
Robust methodology using individual participant data meta-analysis demonstrates that baseline MMP-7 levels predict overall mortality and disease progression in patients with untreated IPF independent of age, gender, smoking status and lung function https://bit.ly/2WlPudQ

Cite this article as: Khan FA, Stewart I, Saini G, *et al*. A systematic review of blood biomarkers with individual participant data meta-analysis of matrix metalloproteinase-7 in idiopathic pulmonary fibrosis. *Eur Respir J* 2022; 59: 2101612 [DOI: 10.1183/13993003.01612-2021].

This single-page version can be shared freely online.

Copyright ©The authors 2022.

This version is distributed under the terms of the Creative Commons Attribution Licence 4.0.

Received: 7 June 2021
Accepted: 21 Aug 2021

Abstract

Background Blood-derived biomarkers have been described extensively as potential prognostic markers in idiopathic pulmonary fibrosis (IPF), but studies have been limited by analyses using data-dependent thresholds, inconsistent adjustment for confounders and an array of end-points, thus often yielding ungeneralisable results. Meta-analysis of individual participant data (IPD) is a powerful tool to overcome these limitations. Through systematic review of blood-derived biomarkers, sufficient studies with measurements of matrix metalloproteinase (MMP)-7 were identified to facilitate standardised analyses of the prognostic potential of this biomarker in IPF.

Methods Electronic databases were searched on 12 November 2020 to identify prospective studies reporting outcomes in patients with untreated IPF, stratified according to at least one pre-specified biomarker, measured at either baseline, or change over 3 months. IPD were sought for studies investigating MMP-7 as a prognostic factor. The primary outcome was overall mortality according to standardised MMP-7 z-scores, with a secondary outcome of disease progression in 12 months, all adjusted for age, gender, smoking and baseline forced vital capacity.

Results IPD was available for nine studies out of 12 identified, reporting outcomes from 1664 participants. Baseline MMP-7 levels were associated with increased mortality risk (adjusted hazard ratio 1.23, 95% CI 1.03–1.48; I^2=64.3%) and disease progression (adjusted OR 1.27, 95% CI 1.11–1.46; I^2=5.9%). In limited studies, 3-month change in MMP-7 was not associated with outcomes.

Conclusion IPD meta-analysis demonstrated that greater baseline MMP-7 levels were independently associated with an increased risk of poor outcomes in patients with untreated IPF, while short-term changes did not reflect disease progression.
